# Gene correction for SCID-X1 in long-term hematopoietic stem cells

**DOI:** 10.1038/s41467-019-09614-y

**Published:** 2019-04-09

**Authors:** Mara Pavel-Dinu, Volker Wiebking, Beruh T. Dejene, Waracharee Srifa, Sruthi Mantri, Carmencita E. Nicolas, Ciaran Lee, Gang Bao, Eric J. Kildebeck, Niraj Punjya, Camille Sindhu, Matthew A. Inlay, Nivedita Saxena, Suk See DeRavin, Harry Malech, Maria Grazia Roncarolo, Kenneth I. Weinberg, Matthew H. Porteus

**Affiliations:** 1 Department of Pediatrics, Division of Stem Cell Transplantation and Regenerative Medicine, Stanford University, Stanford, CA 94305 USA; 2 Department of Bioengineering, Rice University, Houston, TX 77030 USA; 3 Center for Engineering Innovation, University of Texas at Dallas, Richardson, TX 75080 USA; 4 Department of Cellular and Molecular Biosciences, University of California Irvine, Irvine, CA 92697 USA; 5 Laboratory of Host Defenses, National Institutes of Allergy and Infectious Diseases, National Institute of Health, Bethesda, MD 20892 USA; 6 Present Address: University of California Davis, School of Medicine, Sacramento, CA 95817 USA

**Keywords:** Genetic engineering, CRISPR-Cas9 genome editing, Haematopoietic stem cells

## Abstract

Gene correction in human long-term hematopoietic stem cells (LT-HSCs) could be an effective therapy for monogenic diseases of the blood and immune system. Here we describe an approach for X-linked sSevere cCombined iImmunodeficiency (SCID-X1) using targeted integration of a cDNA into the endogenous start codon to functionally correct disease-causing mutations throughout the gene. Using a CRISPR-Cas9/AAV6 based strategy, we achieve up to 20% targeted integration frequencies in LT-HSCs. As measures of the lack of toxicity we observe no evidence of abnormal hematopoiesis following transplantation and no evidence of off-target mutations using a high-fidelity Cas9 as a ribonucleoprotein complex. We achieve high levels of targeting frequencies (median 45%) in CD34^+^ HSPCs from six SCID-X1 patients and demonstrate rescue of lymphopoietic defect in a patient derived HSPC population in vitro and in vivo. In sum, our study provides specificity, toxicity and efficacy data supportive of clinical development of genome editing to treat SCID-Xl.

## Abstract (web summary)

Gene correction in hematopoietic stem cells could be a powerful way to treat monogenic diseases of the blood and immune system. Here the authors develop a strategy using CRISPR-Cas9 and an aAdeno-Associated vVirus(AAV)-delivered IL2RG cDNA to correct X-linked sSevere Ccombined iImmunodeficiency (SCID-X1) with a high success rate.

## Introduction

X-linked sSevere cCombined iImmunodeficiency (SCID-X1) is a primary immune deficiency disorder (PID) caused by mutations in the *IL2RG* gene on the X chromosome. The gene encodes a shared subunit of the receptors for interleukin-2 (IL-2), IL-4, IL-7, IL-9, IL-15, and IL-21. Without early treatment, affected male infants die in the first year of life from infections. Although allogeneic hematopoietic cell transplant (allo-HCT) is considered the standard of care for SCID-X1, it holds significant risks due to potential incomplete immune reconstitution, graft versus host disease (GvHD) and a decreased survival rate in the absence of an human leukocyte antigen (HLA)-matched sibling donor^1^. Because of the selective advantage of lymphoid progenitors expressing normal *IL2RG*, however, only a small number of genetically corrected hematopoietic stem and progenitor cells (HSPCs) are needed to reconstitute T-cell immunity^2,3^. The importance of achieving gene correction in long-term hematopoietic stem cells (LT-HSCs) to achieve sustained clinical benefit is demonstrated by the waning of a functional immune system in patients who do not derive their immune system from LT-HSCs with a wild-type *IL2RG* gene.

Gene therapy is an alternative therapy to allo-HSCT. Using integrating viral vectors, such as gamma-retroviral and lentiviral vectors, extra copies of a functional *IL2RG* gene are semi-randomly integrated into the genome of SCID-X1 patient-derived CD34^+^ HSPCs. This strategy has resulted in both successes and setbacks. While most patients treated with first generation of gene therapy survived and benefited from the therapy, a substantial fraction (>25%) of patients developed leukemia from insertional oncogenesis^4–6^. It is concerning that patients developed leukemia from insertional oncogenesis both early and late, 15 years after transplantation of retroviral-based engineered cells^7^. Constitutive activation of the transgene^8^, the choice of vectors^9^ and specific details of the gene therapy procedure have all been proposed as factors contributing to the risk of leukemia and myelodysplastic syndrome that occurred in several trials for primary immunodeficiency disorders (PIDs) including SCID-X1^10,11^, chronic granulomatous disease (CGD)^12,13^ and Wiskott–Aldrich Syndrome (WAS)^14^. With second-generation self-inactivating (SIN) vectors, multiple SCID-X1 patients have successfully reconstituted T-cell immunity in the absence of early leukemic events^15–17^ with a follow-up of up to 7 years. However, the follow-up of these therapies remains too short to assess the long-term genotoxicity risk of the newer generation vectors, as transformation of T cells growth can take >10 years to manifest^7^.

An alternative to the semi-random delivery of the complementary DNA (cDNA) is to use a targeted genome editing (GE) approach. GE is a means to alter the DNA sequence of a cell, including somatic stem cells, with nucleotide precision. Using homologous recombination-mediated GE (HR-GE), the approach can target a cDNA transgene into its endogenous locus, thereby preserving normal copy number and upstream and downstream non-coding elements that regulate expression^18–20^. The highest frequencies of GE are achieved using an engineered nuclease to create a site-specific double-strand break (DSB) in the cell’s genomic DNA^21,22^. When the DSB is repaired by non-homologous end joining (NHEJ), small insertions and deletions (INDELs) can be created at a specific genomic target site—an outcome that is not generally useful for correcting mutant genes^23,24^. In contrast, when the DSB is repaired by either HR (using a classic gene-targeting donor vector) or by single-stranded template repair (using a single-stranded oligonucleotide (ssODN)), precise sequence changes can be introduced, thereby providing a method to precisely revert disease-causing DNA variants^25^.

Among the multiple GE platforms that use artificial nucleases to generate DSBs^18,26–29^, the CRISPR-Cas9 system has accelerated the field of GE because of its ease of use and high activity in a wide variety of cells. When CRISPR-Cas9 is delivered into primary human cells, including human CD34^+^ HSPCs as a ribonucleoprotein (RNP) complex using fully synthesized single-guide RNA molecules (sgRNAs) with end modifications to protect the guide from exonuclease degradation, high frequencies of INDELs are achieved^30^. Moreover, when the delivery of an RNP complex is combined with delivery of the gene-targeting donor molecule in a recombinant AAV6 (rAAV6) viral vector, high frequencies of homologous-mediated editing in human HSPCs are obtained^25^. The use of rAAV6 donor vectors have been successfully used with other nuclease systems as well, including zinc-finger nucleases (ZFNs) and in other cell types, such as primary human T cells^19,31,32^. Therefore, this HR-GE approach could transform the semi-random nature of viral-based gene therapy to a more controlled and precise strategy. By using AAV6 as a classic gene-targeting donor, in contrast to ssODNs, a full cDNA can be introduced at the endogenous target.

The key challenges in translating GE into medical therapies is attaining clinically relevant targeted integration frequencies into LT-HSCs, attaining functional levels of protein expression, and establishing lack of toxicity derived from the GE approach.

Here, we describe a clinically relevant, selection-free “universal” CRISPR-Cas9-rAAV6 GE methodology that could potentially correct >97% of known *IL2RG* pathogenic mutations. We call this approach “functional gene correction” because it is not directly correcting a mutation but instead is doing so by using the targeted integration of cDNA to functionally correct downstream mutations. Approximately 2–3% of patients with deletions of the gene could not be functionally corrected using this strategy. We demonstrate that a functional, codon-optimized *IL2RG* cDNA can be precisely and efficiently integrated at the endogenous translational start site in CD34^+^ HSPCs of healthy male donors (HD, *n* = 13) or SCID-X1 patients (*n* = 6) at comparable frequencies (median HR = 45%) in both peripheral blood (PB)-derived and umbilical cord blood (CB)-derived CD34^+^ HSPCs. We demonstrate the functionality of the full-length codon-optimized *IL2RG* cDNA by showing that T cells with the cDNA knock-in (KI) retain normal proliferation and signaling response to cytokines. Using transplantation into immunodeficient (NSG) mice, we show that process is both effective (with functional correction of 10–20% of LT-HSCs) and safe (no evidence of abnormal hematopoiesis). The in vivo functional results are based on transplantation of ~21 million *IL2RG* targeted healthy donor CD34^+^ HSPCs and ~7 million *IL2RG* targeted SCID-Xl-HSPCs. We demonstrate high levels of CD34^+^ LT-HSC targeted cDNA integration (10–20%) by showing multi-lineage hematopoiesis derived from these cells using serial transplantation in immunodeficient mice. These results match and exceed the predicted therapeutic threshold determined through a mouse model^19^. Finally, we show no evidence of significant genotoxicity as demonstrated by next-generation sequencing (NGS) and karyotype analysis. Together, this study establishes a pre-clinical proof-of-concept for a safe, precise, and highly efficient GE strategy to potentially cure SCID-X1.

## Results

### Gene correction strategy for *IL2RG* locus in CD34^+^ HSPCs

**Fig. 1:**
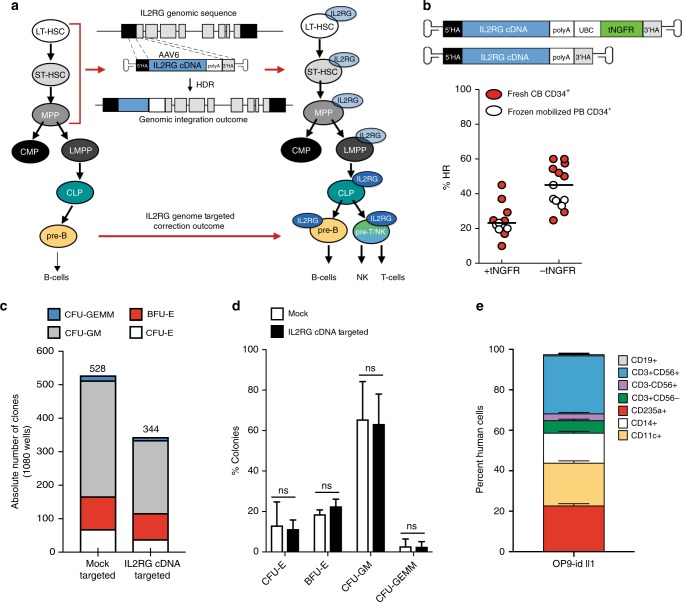
In vitro, medium scale genome targeting at IL2RG locus. **a** Diagram of genomic integration and correction outcomes. **b** Top: schematic of IL2RG corrective donors containing (+tNGFR) or not (–tNGFR) selectable marker. Bottom: IL2RG cDNA targeting frequencies of frozen mobilized peripheral blood CD34^+^HSPCs (white circles) or freshly purified cord blood male-derived CD34^+^HSCPs (red circles) derived from medium scale (1.0 × 10 ^6^) genome targeting and measured at day 4. Absolute targeting frequencies measured by ddPCR. Median: 23.2% (+tNGFR, *n* = 11 biological replicates), median 45% (–tNGFR, *n* = 13 biological replicates). **c** Single cell-based methylcellulose assay from mock targeted (nucleofected only) or IL2RG cDNA targeted (–tNGFR donor) CD34^+^HSPCs. Absolute number of clones are shown (*n* = 3 biological replicates). **d** Fraction of the total for each type of colony scored. **e** Gene correction outcome of SCID-X1 patient 2 derived CD34^+^HSPCs. Shown is the multi-lineage differentiation using OP9-idll1 in vitro system (*n* = 23 wells). No growth was derived from uncorrected CD34^+^cells. LT-HSPCs long-term hematopoietic stem cells, ST-HSC short term hematopoietic stem cells, MPP multi-potent progenitor, CMP common myeloid progenitor, LMPP lymphoid multi-potent progenitor, CLP common lymphoid progenitor, HSPCs hematopoietic stem and progenitor cells, ddPCR droplet digital digital droplet PCR. Mean ± s.e.m.; ns not specific (Welch’s *t-*test). Source data are available in the Source Data file

SCID-X1 is caused by pathogenic mutations spanning the entire *IL2RG* gene. Therefore, we developed a gene-targeting strategy by integrating a complete cDNA at the endogenous *IL2RG* translational start site (Fig. 1a, central panel) that would correct the vast majority (~97%) of known SCID-X1 pathogenic mutations and ensure regulated endogenous expression in CD34^+^ HSPCs derived progeny. By achieving efficient integration frequencies in the genome of CD34^+^ LT-HSCs, our approach could ensure life-long therapeutic benefits for the patient (Fig. 1a, right schematic).

We screened seven different sgRNAs (single guide RNAs) for activity in exon 1 of the *IL2RG* gene (Supplementary Fig. 1a) and selected sg-1, previously described^30^, as the best candidate because of the location of the DSB it creates (one nucleotide downstream from the translational start site), on-target INDEL frequencies (92.9% ± 0.6, mean ± s.e.m) (Supplementary Fig. 1b) and for high cellular viability >80% (Supplementary Fig. 1c). We found that a truncated sgRNA^33^ of 19 nucleotides (19 nt) gave >90% INDEL frequencies (equivalent to the full 20 nt sgRNA) (Supplementary Figs. 1–3). NGS (Supplementary Fig. 1e) further corroborated the INDELs obtained by TIDE analysis^34^. We used the 19 nt gRNA at a medium scale process (1 million cells per electroporation) throughout the remaining experiments.

We designed a codon-optimized *IL2RG* cDNA functional correction donor with homology arms centered on the sg-1 guide sequence and cloned into an AAV6 vector both with and without a selectable marker (truncated nerve growth factor receptor (tNGFR) driven by the Ubiquitin C promoter (Supplementary Figs. 4a, b) (Fig. 1b, top panel). The efficiency of genome targeting integration was determined in both frozen mobilized PB (mPB) and in freshly isolated CB-derived CD34^+^ HSPCs from healthy male donors (Fig. 1b). We observed a median gene-targeting frequency of 23.2% (range 9.9–45.0%) for the +tNGFR donor and 45.0% (range 24.7–60.0%) for the –tNGFR *IL2RG* donor (Fig. 1b, bottom panel), as measured by Digital Droplet Droplet Digital PCR (ddPCR) **(**Supplementary Figs. 5a–f**)**. As the selection-free cassette gave high frequencies of targeted integration, we concluded that a selection marker was not necessary because it would create a simpler cell manufacturing process for cells that would also have a selective advantage.

To determine the myeloerythroid differentiation potential of *IL2RG* cDNA genome targeted CD34^+^ HSPCs, we performed methylcellulose assays. After Cas9/gRNA-rAAV6-CRISPR-Cas9-AAV6 based *IL2RG* cDNA targeting, HSPCs were single-cell plated in a 96-well methylcellulose plates and scored for colony formation at day 14. Although the number of colonies was reduced by ~35% in *IL2RG* cDNA targeted samples compared with mock-targeted HSPCs (where neither the sgRNA nor the donor were introduced) (Fig. 1c), the distribution of types of colony-forming units (CFUs) was the same from *IL2RG* cDNA targeted HSPCs and mock-targeted HSPCs, including CFU-GEMMs (granulocytes, erythrocytes, monocytes, megakaryocytes), without any lineage skewing (Fig. 1d). Genotyping of colonies confirmed that *IL2RG* cDNA targeted derived-colonies (*n* = 344) showed an overall targeting frequency of 45.7% ± 2.4 (mean ± s.e.m.) (Supplementary Fig. 6). Bi-allelic modification is not relevant as the cells were derived from male donors and have a single X chromosome. In sum, the in vitro differentiation assay of targeted *IL2RG* cDNA CD34^+^ HSPCs demonstrated no perturbation of the myeloerythroid differentiation potential.

To assess the hematopoietic differentiation potential of the codon-optimized *IL2RG* cDNA donor, we used the OP9-idll1 stromal cell line in vitro system. In this system, a lentiviral vector confers the doxycycline (DOX)-inducible expression of the Notch ligand Dll^35^. In the presence of a cocktail of cytokines permissive for myeloerythroid and lymphoid differentiation, multi-potent human CD34^+^ HSPCs will generate only myeloerythroid and B-cell lineage before induction of dll1 expression, but becomes permissive for T and NK-cell generation in the same well after addition of DOX to induce dll1 expression. CD34^+^ HSPCs derived from frozen mPB of SCID-X1 patient (delA;M145fs—patient 2) were gene targeted (functionally corrected) using the CRISPR-Cas9-AAV6 platform. The total number of cells per well derived from the *IL2RG* cDNA targeted cells was markedly increased, compared with that of mutant cells, indicating a growth dependence on functional IL-7 and IL-15 receptors, for which *IL2RG* is an essential subunit^36^. Following DOX-mediated dll1 expression, no further growth of mutant CD34^+^ HSPCs was detected on OP9-idll1 stromal cells. In contrast, the *IL2RG* cDNA targeted cells continued to expand in myeloerythroid compartment in addition to the development of B (CD19^+^), T (CD3^+^CD56^-^), NK (CD3^−^CD56^+^), and TNK (CD3^+^CD56^+^) progeny progenitors (Fig. 1e, Supplementary Fig. 7). The OP9-idll1 in vitro system is known to generate more CD3^+^CD4^+^ over CD3^+^CD8^+^ cells expressing cell surface markers^37^. A CD3^+^CD56^+^ (TNK cell) population was generated from our genome corrected SCID-X1 patient-derived CD34^+^ HSPCs further demonstrating the range of lymphoid reconstition that can arise following ex vivo gene editing correction of the *IL2RG* gene from patient-derived cells^38^. These experiments demonstrate the functional correction of the *IL2RG* gene from patient-derived CD34^+^ HSPCs necessary for lymphoid development.

### Hematopoietic reconstitution from *IL2RG* cDNA targeted HSPCs

**Fig. 2:**
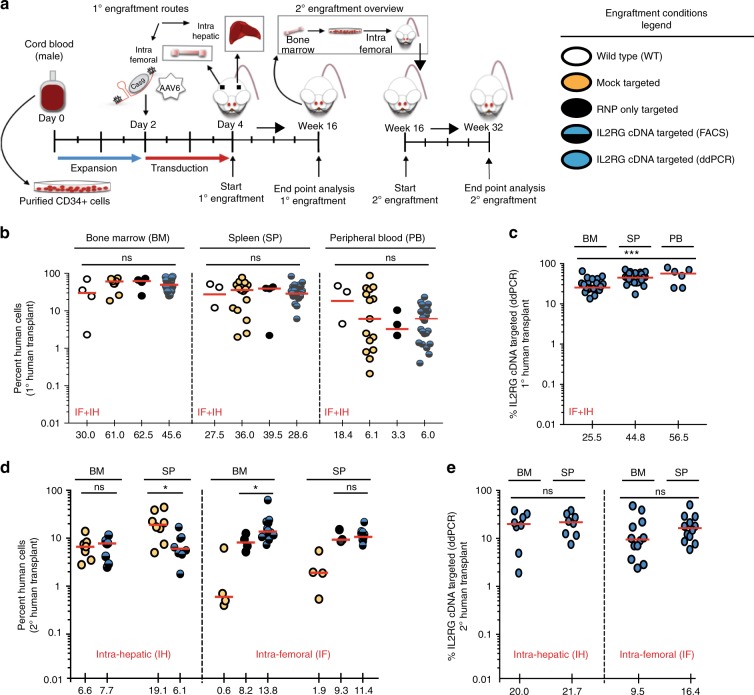
Normal hematopoietic reconstitution from *IL2RG* cDNA targeted CD34^+ ^HSPCs. **a** Timeline of primary (1°) and secondary (2°) human transplants into sub-lethally irradiated NSG mice. CD34^+ ^HSPCs are derived from umbilical cord blood of healthy male donors. Adult mice transplanted intra-femoral (IF) with either WT CD34^+^HSPCs (white circles) or mock targeted (yellow circles) or RNP only (black circles) or un-selected *IL2RG* cDNA targeted (blue-black circles) HSPCs. Three – 4 days old NSG pups transplanted intra-hepatic (IH) with either mock or *IL2RG* targeted HSPCs. **b** Combined IF and IH human cells engraftment (hCD45^+^HLA A-B-C^+^) 16 weeks after 1° human transplant into indicated organs. **c** %*IL2RG* cDNA targeted HSPCs within human graft in indicated organs,  quantified by ddPCR. BM (*n* = 24 mice), SP (*n* = 24 mice), PB (*n* = 6 mice) (***p = 0.0008, one-way ANOVA). **d** Percent human engraftment in indicated organs as in (**b**) 16 weeks post 2° human CD34^+ ^HSPCs transplant into adult NSG mice. *p-value SP-IH = 0.025, *p-value BM-IF = 0.043 (Welch’s *t*-test). **e** %*IL2RG* targeted HSPCs quantied by ddPCR 32 weeks after engraftment. Median shown. BM bone marrow, SP spleen, PB peripheral blood. Source data are available in the Source Data file

**Fig. 3:**
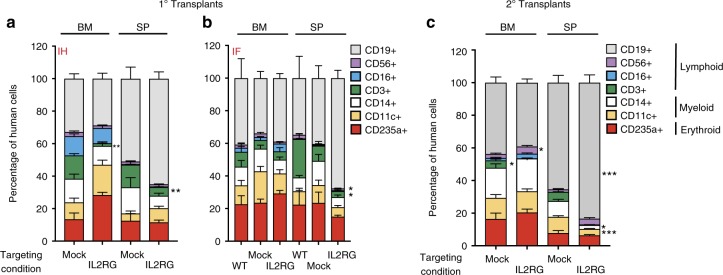
Normal multi-lineage development from *IL2RG* cDNA targeted in the LT-HSC population. **a** Percent cellular composition of the lymphoid, myeloid and erythroid lineage derived from IH 1° human engraftment, shown in indicated organs and targeting conditions. CD3^+ ^BM: **p = 0.0017, CD3+SP: **p = 0.007 (Welch’s *t*-test). **b** Same as (**a**) but IF transplant analysis. CD3^+ ^SP: *p = 0.023, CD56^+ ^BM: *p = 0.015 (Kruskal–Wallis test). **c** Percent cellular composition of the lymphoid, myeloid and erythroid lineage derived from secondary transplants. Data shown are combined IH and IF primary transplants. CD3^+ ^BM: *p = 0.015, CD56^+^: *p = 0.025, CD19^+^ SP: ***p = 0.0002, CD14^+^ SP: *p = 0.0112, CD11c^+^ SP: ***p = 0.0004. LT long term. Error bars: mean ± s.e.m. Source data are available in the Source Data file

To further assess the toxicity and efficacy of our HR-GE system, we evaluated the in vivo engraftment and multi-lineage hematopoietic reconstitution of *IL2RG* cDNA targeted HSPCs in immunodeficient NSG mice. Following ~4 days of ex vivo manufacturing, *IL2RG* cDNA targeted and different control cells were transplanted either by intra-hepatic (IH) injection into sub-lethally irradiated 3- to 4-day-old NSG pups or by intra-femoral (IF) injection into 6- to 8-week-old NSG mice. The IH system has previously been shown to be superior for assessment of human lymphopoiesis^39^. An experimental schema is shown (Fig. 2a, primary engraftment panel). For primary engraftment studies, a total of 19.3 million cells, derived from three different healthy male CB CD34^+^ HSPCs were transplanted into a total of 47 mice (Fig. 2b). The kinetics of primary human engraftment was monitored at weeks 8 and 12 in bone marrow (BM) aspirates and PB samples. At week 16, end point analysis was carried out on total BM, spleen (SP), and PB samples. High human engraftment levels - as shown by hCD45^+^ HLA-ABC^+^ double positive staining, blue/black circles – were obtained with no statistical difference between the *IL2RG* cDNA targeted and control cells — WT, mock, or RNP (Fig. 2b, Supplementary Figs. 8, 9). Transplanted *IL2RG* targeted HSPCs showed a median human engraftment level of 45% in BM (*n* =24), 28% in SP (*n* = 24), and 6% in PB (*n* = 24) (Fig. 2b**)**. The targeted integration frequency of the *IL2RG* cDNA was 25.5% in BM (*n* = 24), 44.8% in SP (*n* = 24), and 56% in PB (*n* = 6) at week 16 post engraftment (Fig. 2c). Multi-lineage reconstitution was achieved from both mock and *IL2RG* cDNA targeted cells in both the BM and SP samples of transplanted mice (Fig. 3a).

In human cells not targeted with the cDNA correction cassette, the frequency of INDELs was >90% in the IH engrafted *IL2RG* targeted cells at weeks 8, 12, and 16 with an INDEL spectrum of +1, −11, and −13 (all inactivating mutations) **(**Supplementary Fig. 10**)**. In sum, the engraftment of selection-free *IL2RG* cDNA targeted CD34^+^ HSPCs derived from healthy male donors demonstrate the ability to give rise to normal hematopoiesis. As >90% of the non-gene targeted human cells have inactivating INDELs in the *Il2RG* gene, it is likely that the T and NK cells seen in the mice are derived from gene targeted CD34^+ ^HSPCs. The paucity of these cells in the mice, however, precluded definitive molecular analysis.

### *IL2RG* cDNA genome targeting of LT-HSCs

The editing of LT-HSCs would provide the long-term maintenance of T-cell function in patients. We performed secondary transplantation studies to assess the robustness of our CRISPR-Cas9-AAV6 genome targeting platform in editing LT-HSCs. CD34^+^ HSPCs were isolated from total BM of *IL2RG* cDNA targeted HSPCs (from both primary IH or IF engraftments at week 16). Following overnight culturing, secondary transplants were carried out in sub-lethally irradiated 6- to 8-week-old NSG mice (Fig. 2a). At 16 weeks following the secondary transplant, end point analysis — totaling 32 weeks of engraftment into immunodeficient mice — a median human chimerism level (hCD45^+^/ HLA-ABC^+^ double positive cells) of *IL2RG* cDNA targeted cells ranged from 7.7% to 13.8% (BM) and 6.1% to 11.4% (SP) (Fig. 2d). The median targeted integration frequencies of the *IL2RG* cDNA donor was 9.5% or 20% (BM) and 16.4% or 21.7% (SP) (Fig. 2e). Fluorescence-activated cell sorting (FACS) plots showing BM human engraftment levels from mice injected with cells derived from both conditions are shown (Supplementary Figs. 11, 12). Analysis in secondary transplants showed multi-lineage hematopoietic reconstitution with no evidence of abnormal hematopoiesis thus providing further evidence of efficacy and safety (Fig. 3c).

A summary of the *IL2RG* cDNA targeted engrafted cells is shown in Tables 1 and 2. We report that 20 and 9.5% of human cells in the BM derived from IH-IF and IF-IF secondary xenotransplantation experiments, respectively, retain the codon-optimized *IL2RG* cDNA donor integration, demonstrating a clinically significant level of correction of CD34^+^ LT-HSCs. Moreover, our median frequencies of *IL2RG* cDNA targeted in LT-HSCs significantly exceeds those reported by other groups, notably Genovese et al.^20^ (ZFNs), Schiroli et al.^19^ (ZFNs), and Dever et al.^25^ (Cas9 RNP) where the percent of HR-GE cells was <5% of the human cells engrafted. These results, therefore, represent the first evidence of high frequencies of HR-GE in LT-HSCs using the CRISPR-Cas9 system. No tumors or abnormal hematopoiesis were observed in any mice that were transplanted with genome-modified cells (RNP or *IL2RG* cDNA targeted). Collectively, our primary and secondary transplantation results validate the robustness, effectiveness and lack of genotoxicity of our *IL2RG* cDNA genome targeting approach and strongly supports its advancement towards clinical translation.

**Table 1:** Summary of total number of cells and mice injected per condition for primary (1°) and secondary (2°) transplants

Genome editing condition transplanted into NSG mice	1° Human transplant studies total nr. of cells injected per condition	1° Human transplant studies total nr. of mice per condition (end point)	2° Human transplant studies total nr. of cells injected per condition	2° Human transplant studies total nr. of mice per condition (end point)
WT	2.0 × 10^6^	4	n/a	n/a
Mock targeted	5.7 × 10^6^	15	4.3 × 10^6^	12
RNP only targeted	2.0 × 10^6^	4	1.5 × 10^6^	4
IL2RG cDNA targeted	9.7 × 10^6^	24	8.0 × 10^6^	20
Total	19.3 × 10^6^	47	13.8 × 10^6^	36

**Table 2:** *IL2RG* cDNA genome targeted frequencies pre- and pos-t transplant

Time of genome editing quantification (ddPCR)	% Functionally corrected cells (BM) IH engraftment	% Functionally corrected cells (BM) IF engraftment
Prior to 1° transplant	55% of CD34^+^ HSPCs	44% CD34^+^ HSPCs
Prior to 1° transplant (16 weeks)	28% of CD34^+^ HSPCs	26% of CD34^+^ HSPCs
2° Transplant (32 weeks)	20% of CD34^+^ HSPCs	9.5% of CD34^+^ HSPCs

Percent homologous direct repair (HDR) as measured by ddPCR at the indicated time points. CD34^+^ HSPCs are derived from healthy male donors. Median shown. 1° transplant: IH (*n* = 10), IF (*n* = 14). 2° transplant: IH (*n* = 8), IF (*n* = 12)

### In vivo rescue of lymphopoiesis

**Fig. 4:**
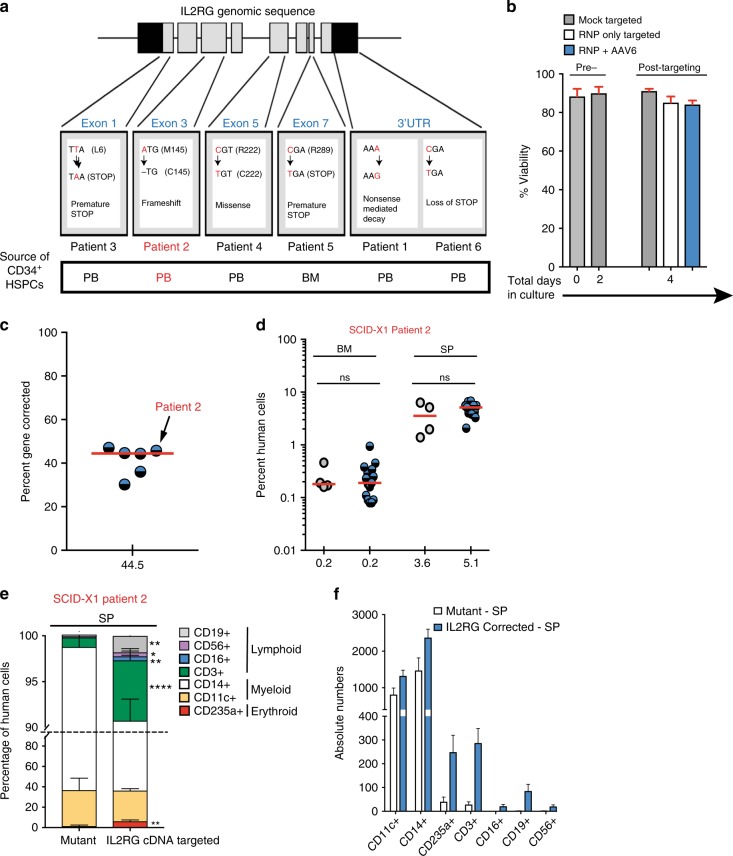
In vivo rescue of SCID-X1 mutation. **a** Genomic mapping and description of SCID-X1 mutations. **b** Percent viability determined at indicated days pre- and post- targeting. Mock (nucleofected only), RNP (nucleofected with RNP only), RNP+AAV6 (nucleofected with RNP and transduced with AAV6-based *IL2RG* corrective donor). Shown is data for mobilized peripheral blood CD34^+^ HSPCs (*n* = 5). **c** Medium scale (1.0 × 10^6^ cells) *ex vivo* genome targeting frequencies of frozen mobilized peripheral blood SCID-X1, at day 2 (blue-black circles, *n* = 6). Arrow shows 45% genome targeting of SCID-X1 patient 2 derived CD34^+ ^HSPCs. **d** Human cells engraftment analysis at week 17 after intra-hepatic (IH) delivery of *IL2RG* cDNA targeted (blue-black circles, *n* = 15) or mutant CD34^+^ HSPCs (gray circles, *n* = 4). **e** Percent cellular composition of the lymphoid, myeloid, and erythroid lineage derived from *IL2RG* corrected or mutant CD34^+^ HSPCs. CD3^+^: ****p < 0.0001, CD56^+^: *p = 0.0146, CD16^+^: **p = 0.0013, CD19^+^: **p = 0.0015, CD235a^+^: **p = 0.0022 (Welch’s *t-*test). RNP ribonuclearprotein. **f** Absolute numbers derived from (**e**). Source data are available in the Source Data file

We investigated whether our gene-targeting approach was reproducible and efficient in SCID-X1 patient-derived CD34^+^ HSPCs. We edited CD34^+^ HSPCs from six different SCID-X1 patients with a variety of different pathologic mutations (Fig. 4a). Five of the six samples were PB-derived CD34^+^ HSPCs. We achieved high viability (>80%, *n* = 5) with the CRISPR-Cas9-AAV6 system in the patient-derived cells and high gene-targeting (median 44.5 with range of 30.1– 47.0%, *n* = 6), a frequency comparable to healthy donor CD34^+^HSPCs (45%, *n* = 13) (Figs. 4b, c). A total of 7.3 million edited CD34^+^ HSPCs derived from patients 1, 2, and 3 were engrafted into 29 NSG pups. Human chimerism was measured at week 16 following IH engraftment, with no statistically significant differences between unmodified and *IL2RG* cDNA targeted cells, both in the BM and SP samples obtained from mice transplanted with SCID-X1 patients 1 and 2 derived CD34^+^ HSPCs (Fig. 4e and Supplementary Fig. 13). A statistically significant difference was observed only in BM samples derived from SCID-X1 patient 3 engraftment (**p = 0.0073, Holm–Sidak test) (Supplementary Fig. 13). Importantly, only the codon-optimized *IL2RG* cDNA (not mutant allele) was detected in the SP of mice (*n* = 8) engrafted with SCID-Xl patient 2 corrected CD34^+^ HSPCs (Table 3), consistent with the survival advantage that a cell with a corrected *IL2RG* gene has. Multi-lineage analysis of SP samples derived from mice engrafted with *IL2RG* cDNA targeted SCID-Xl mPB CD34^+^ HSPCs derived from patient 2 showed that significant levels of erythroid, myeloid, and lymphoid lineages were established (Figs. 4e, f). Gene corrected cells from both patients 1 and 3 showed high levels of engraftment following transplantation in both BM and SP (Supplementary Fig. 13). This work is the first to show in vivo rescue of the lymphoid lineage in a SCID-X1 patient-derived CD34^+^ HSPCs. In sum, these transplantation studies demonstrated that *IL2RG* cDNA targeted CD34^+^ HSPCs can engraft and rescue the SCID-X1 phenotype, as demonstrated by multi-lineage reconstitution both in vitro and in vivo. We observed no abnormal hematopoiesis in mice transplanted with HR-GE patient-derived cells providing further evidence for the safety of the process.

**Table 3:** Summary of SCID-X1 patients’ derived CD34^+^ HSPCs transplants

SCID-X1 patients 1–3	Total nr. of cells injected per condition	Total nr. of mice per group
SCID-X1 mutant CD34^+^ HSPCs	3.25 × 10^6^	13
IL2RG cDNA targeted CD34^+^ HSPCs	7.25 × 10^6^	29

### Signaling and proliferation of *IL2RG* cDNA targeted T cells

**Fig. 5:**
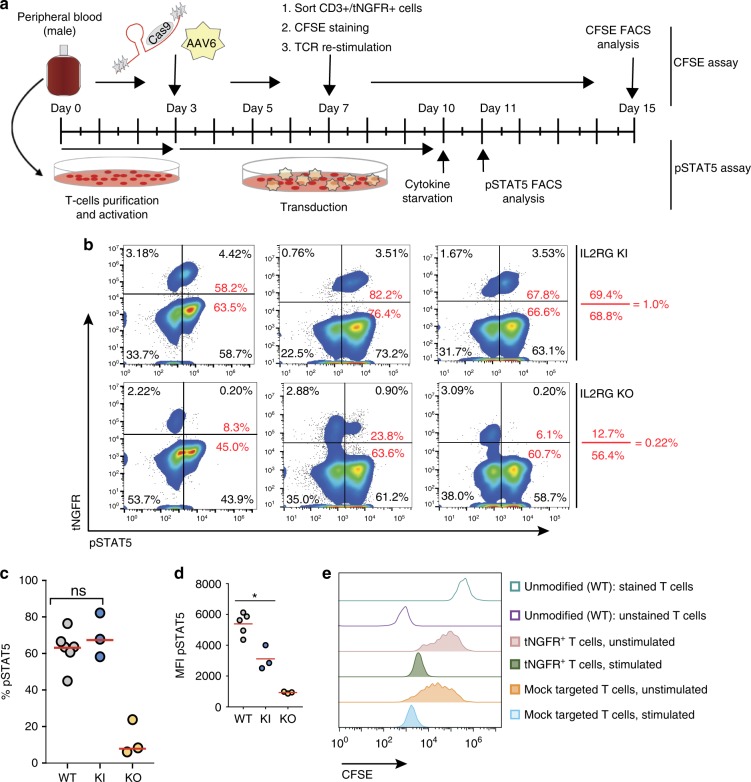
Evaluation of IL-2 receptor function in *IL2RG* cDNA targeted T cells. **a** Schematic of signaling (pSTAT5—bottom) and proliferation (CFSE—top) in vitro assays. **b** pSTAT5 assay derived FACS plots. Top: healthy male-derived T cells genome targeted with *IL2RG* cDNA tNGFR (KI) or with tNGFR+ only cassette integrated at the *IL2RG* endogenous locus (KO). In red are the percent of double positive *IL2RG*-tNGFR^+^pSTAT5^+^[4.42%/(4/42% + 3.18%)]×100. We compare 58.2% cells (IL2RG targeted T cells) with 58.7% (*IL2RG* from WT T cells), (*n* = 3 biological replicates). **c** Quantification of IL-2R signaling through phosphoSTAT5 pathway. **d** pSTAT5 MFI for WT, KI, and KO experiments from (**b**) *p* = 0.02, Welch’s *t*-test. WT T cells (gray circles, *n* = 6), *IL2RG* KI (blue circles, *n* = 3) and *IL2RG* KO (orange circles, *n* = 3). **e** Proliferation profile of CFSE labeled, TCR stimulated *IL2RG* cDNA tNGFR+ sorted or mock-targeted T cells. Mock-targeted T cells are WT T cells cultured for the same amount of time as the tNGFR+ targeted cells and have been nucleofected in the absence of RNP or absence of transduction with AAV6. Shown FACS analysis at days 2, 4, 6, and 8. pSTAT5 phosphorylated STAT5, CFSE carboxyfluorescein succinimidyl ester, KI knocked in, KO knocked out, tNGFR truncated nerve growth factor receptor, IL-2 interleukin 2. Source data are available in the Source Data file

To assess the receptor function and signaling in progenitor cells in which the gene is express through the targeted integration of a codon-optimized cDNA into the translational start site of the endogenous locus, we evaluated the proliferation and signaling activity of HR-GE human T lymphocytes derived from adult healthy male donors. Mature T cells depend on proper *IL2RG* expression and signaling through *IL2RG*-containing receptors, e.g., IL-2R, to promote proliferation and differentiation^40^. Activation of T cells by CD3/CD28 antibodies leads to a rapid induction of IL-2 cytokine, which in turn signals though the IL-2R. Subsequent phosphorylation of tyrosine residues on the cytoplasmic domains of the receptors initiates a cascade of events that phosphorylate and activate the signaling transducers and activators of transcription 5 (STAT5) proteins. Therefore, we assessed the levels of pSTAT5 in *IL2RG* cDNA targeted T cells, where the *IL2RG* cDNA donor contained tNGFR selectable marker (Fig. 5a). Intracellular staining for pSTAT5 from *IL2RG* cDNA targeted T cells (Fig. 5b) and levels of pSTAT5 (ratio of tNGFR^+^pSTAT5^+^ double-positive cells to that of tNGFR^+^ only cells, marked red) was demonstrated to be comparable to that of unmodified normal T cells 69.3 ± 7.0 vs 67.7 ± 4.4 (mean ± s.e.m.), respectively (Fig. 5c). As expected, knocking-out (KO) the *IL2RG* locus with an *IL2RG* targeted donor expressing only tNGFR, significantly reduced the levels of pSTAT5 (12.7 ± 5.6; mean ± s.e.m.) (Fig. 5b). We analyzed the MFI of the pSTAT5 level in WT, KI, and KO cells (Fig. 5c) and found that the KO cells had an extremely low pSTAT5 MFI (as expected), whereas the KI cells had pSTAT5 MFI (mean fluorescence intensity) that was ~50% of the wild-type cells. This lower signaling did not compromise lymphocyte development (Figs. 1–4**)** nor proliferation (Fig. 5d). The KI cells did not have higher signaling, which has been hypothesized as a risk factor for transformation.

To demonstrate that the genome edited IL-2R is permissive for proliferation upon engagement of IL-2 cytokine, we quantified the levels of proliferation of *IL2RG* cDNA targeted T-cell following T-cell receptor (TCR) stimulation. A carboxyfluorescein succinimidyl ester (CFSE) dilution assay was used to measure whether targeted insertion of the codon-optimized cDNA could support T-cell proliferation. Loss of CFSE signal occurs when cells proliferate as the dye dilutes from cell division. An overview of the assay is shown (Fig. 5a). In our experimental settings, we observed similar proliferation profile in tNGFR^+^ T cells (marking cells in which the *IL2RG* cDNA had been KI) compared with mock-targeted cells (Fig. 5e). We note that the “unmodified” cells had not undergone prior bead stimulation and so remained quiescent while the targeted and mock cells had undergone prior bead stimulation and thus there was residual proliferation without re-stimulation in those cells giving the broader peak. Overall, our data demonstrate that the genomic integration of an *IL2RG* codon diverged cDNA at the start site of the endogenous locus preserves normal signaling and proliferation of human T cells.

### Off-target and karyotype analysis

**Fig. 6:**
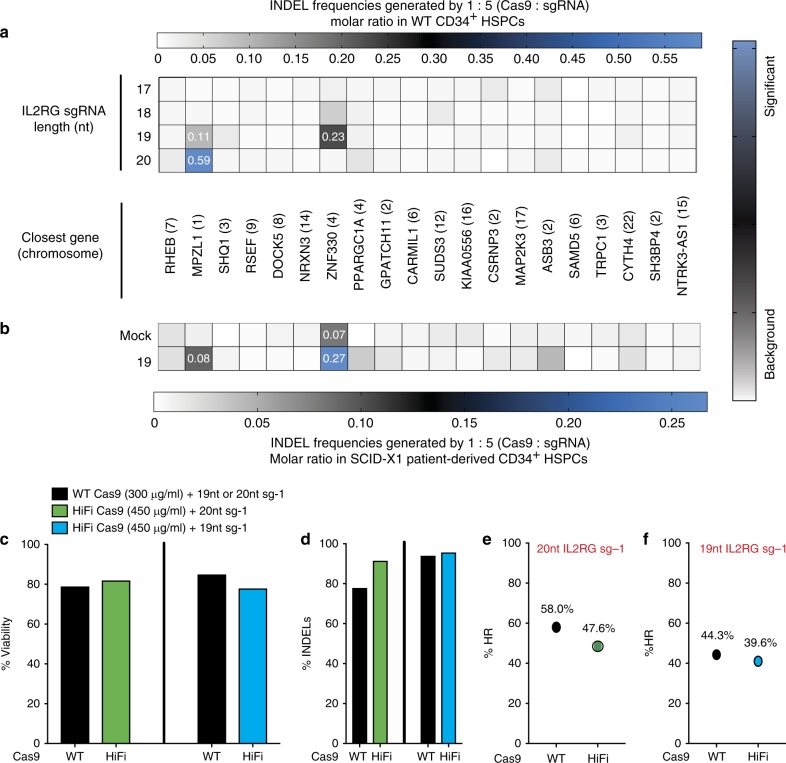
Genome specificity of *IL2RG* sgRNA guide. **a** Heat map of on-target INDEL frequencies quantied by NexGen-Seq at COSMID identified putative on-target locations from healthy CD34^+ ^HSPCs. Levels of NHEJ induced by 20 nt *IL2RG* sgRNA and truncated 19 nt, 18 nt and 17 nt pre-complexed with WT Cas9 protein at 5:1 molar ratio. **b** Heat map as in (**a**) of on-target INDEL frequencies derived from 19 nt IL2RG sg-1 in the genome of CD34^+ ^HSPCs SCID-X1 patient 1 derived cells. **c** Percent viability at day 4 of SCID-X1 patient-derived CD34^+ ^HSPCs nucleofected with either wild-type (WT) or high-fidelity (HiFi) SpCas9 protein pre-complexed with either the 20 nt or the 19 nt IL2RG sg-1 (*n* = 1). **d** Percent INDELs measured by TIDE at day 4 in cells as in (**c**) using WT or HiFi Cas9 protein pre-complexed with the 20 nt *IL2RG* sg-1 (green bars) or 19 nt IL2RG sg-1 (blue bars). **e** Percent *IL2RG* cDNA targeting (% HR) as measured by ddPCR at day 4 in cells as in (**c**) generated by either WT or HiFi Cas9 protein pre-complexed with the 20 nt *IL2RG* sg-1 or (**f**) 19 nt *IL2RG* sg-1. Source data are available in the Source Data file

We investigated the specificity of the dsDNA break generated by the CRISPR–Cas9 RNP complex, which could be a potential source of genotoxicity. The off-target activity of the full-length 20 nt and three truncated versions (19 nt, 18 nt, and 17 nt) of sg-1 guide were assessed at 54 different potential sites predicted by either Guide-Seq in U20S cells^41^ or bioinformatically COSMID^42^ (Fig. 6a). The analysis was performed in both healthy (Fig. 6a) and patient-derived CD34^+^ HSPCs (Fig. 6b) to assess the specificity of the sg-1 gRNA. At the three sites identified by Guide-Seq analysis, there was no evidence of off-target INDELs. In the 51 sites identified by COSMID, only two showed evidence of off-target INDELs, both at levels <1% (Fig. 6a). We detected INDEL frequencies using the 20 nt sg-1 of 0.59%, in an intron of myelin protein zero-like 1(*MPZL1*), a cell surface receptor gene involved in signal transduction processes. The 19 nt sg-1 induced a lower frequency of off-target INDELs 0.11% (Fig. 6a and Table 4). We also analyzed the INDEL frequencies of potential off-target sites in genome edited CD34^+^ HSPCs derived from SCID-Xl patient 1 in which the cells were edited using the 19 nt sg-1 (Fig. 6b). We found INDEL frequencies of 0.08% at *MPZL1* and 0.27% at the *ZNF330* site (intergenic and >9 kb from the nearby gene, respectively) (Table 4). Off-target activities of sg-1 guides, WT (20 nt) and truncated (19 nt), were further assessed in the context of a high-fidelity (HiFi) Cas9^43^ in SCID-X1 CD34^+^ HSPCs. The viability, INDELs, and *IL2RG* cDNA targeting frequency (%HR) were all equivalent (Fig. 6c) and editing frequencies (% INDELs) (Fig. 6d) were comparable between WT and HiFi Cas9 (Figs. 6c–f). Using the 20 nt and 19 nt gRNA combined with the HiFi Cas9, however, resulted in no detectable INDELs (“background” Table 4) at the two sites for which there was low but detectable INDEL frequency using WT Cas9.

**Table 4:** Summary of *IL2RG*sgRNA off-target INDEL frequency analysis

Gene name	Guide Seq	COSMID	Chromosome location	Features	Expression in HSCs^a^	U2OS (plasmid)	WT CD34^+^ 20 nt RNP (WT Cas9)	SCID-X1 CD34^+^ 19 nt RNP (WT Cas9)	SCID-X1 CD34^+^ 20 nt RNP (HiFi Cas9)	SCID-X1 CD34^+^19 nt RNP (HiFi Cas9)
IL2RG			X	Exon	Yes	81.1%	81.7%	91.7%	94.1%	97.6%
LIN01287	✓		7	Intergenic	Data not available	Background	Background	Background	Not sequenced	Not sequenced
MPZL1		✓	1	Intron	Yes	1.1%	0.1%	0.1%	Background	Background
SHQ1	✓	✓	3	Intron	Yes	1.5%	Background	Background	Not sequenced	Not sequenced
SMYD3	✓		1	Intron	Yes	4.2%	Background	Background	Not sequenced	Not Sequenced
ZNF330		✓	4	Intergenic	Data not available	Background	0.23%	0.27%	Background	Background

^a^Expression determined by www.biogps.org and Gene Expression Commons

To further assess whether genomic instabilities, particularly translocations, were generated by the - CRISPR-Cas9-AAV6 based process, we performed karyotype analysis on CB-derived CD34^+^ HSPCs from healthy male donors. We chose to run karyotype analysis over PCR-based translocation assays because we have previously found that the frequency of translocations in CD34^+^ HSPCs when two on-target breaks (with INDEL frequencies of >80%) was 0.2–0.5%^44^. The probability that there is a translocation between the on-target break and break that has an INDEL frequency of <0.1% is exceedingly low. Whole chromosomal analysis was performed on ≥20 cells from the different conditions (WT, mock only, RNP only, AAV6 only and RNP+AAV6). The analysis confirms absence of any chromosomal abnormalities in 20 out of 20 untreated or mock treated cells, 40 out of 40 RNP only or RNP treated with rAAV6 cells, and from 40 out of 40 cells treated with rAAV6 only (Supplementary Fig. 15). Finally, we performed γH2AX and relative survival assays in K562 and 293T cells lines, respectively, to determine and compare the levels of DNA damage and toxicity induced by ZFN, TALEN, and CRISPR-Cas9 nucleases that all target the *IL2RG* gene (Supplementary Fig. 16). The CCR5 ZFNs were first described in Perez et al.^45^ and subsequently used to clinically and to modify CD34^+^ HSPCs^24,46,47^. The nucleases targeting the *IL2RG* gene were described previously in Urnov et al.^26^ (ZFNs) and Hendel et al.^48^ (ZFNs, TALENs, and CRISPR-Cas9). The  CRISPR-Cas9 nuclease generated the lowest levels of toxicity by showing fewer γH2AX foci and higher percent survival of human cells overexpressing each nuclease^49^ highlighting the notion that standard TALEN and ZFN nuclease platforms are less specific than CRISPR-Cas9.

In conclusion, our off-target analysis confirms that high specificity and activity is achieved using the *IL2RG* CRISPR-Cas9-AAV6 HR-GE system described here.

## Discussion

Currently there are numerous GE-based clinical trials in USA and China, none of which are for the treatment of PIDs (clinicaltrials.gov). There have been a number of proof-of-concept GE studies to explore the feasibility and safety of using a HR-mediated approach to correcting pathologic mutations in the *IL2RG* gene as a path to developing an auto-HSPC-based therapy for SCID-X1^19,20,26,50–54^. In particular, a recent study by Schiroli et al.^19^, in the process of developing a clinical translation GE for SCID-Xl in CD34^+^ HSPCs, designed a ZFN GE-based platform to integrate a full *IL2RG* cDNA at intron 1 delivered by integration-defective lentiviral vector or rAAV6. They were able to generate ~40% INDELs and ~10% HR frequencies in WT CD34^+^ HSPCs, with targeted integration frequencies of ~25% in one SCID-Xl patient-derived CB CD34^+^ HSPCs. Notably, Schiroli et al.^19^ performed only one experiment combining CRISPR-Cas9 with AAV6 and almost all of their data, including the engraftment data were from ZFN-modified cells. Our work represents significant progress for CRISPR-Cas9-based approaches as we not only demonstrate high levels of engraftment of targeted cells in LT-HSCs (up to 20%), we also demonstrate targeting efficiencies and engraftment of efficiencies in patient-derived CD34^+^ HSPCs that exceed the LT-HSC threshold of 10%. This high levels of genomic editing of LT-HSCs has not been previously reported and demonstrates with advances in technology, significant biologic improvements are possible with clinically relevant quantitative metrics being met. This level of correction is likely to be curative based on both animal studies^19^, from patients who had spontaneous reversion mutations in progenitor cells and from human gene therapy clinical trials. In the gene therapy clinical trials for SCID-X1, immune reconstitution was achieved with as little as 1% of the cells having gene transfer^3^ or from vector copy numbers of only 0.1 in the blood^2^. Our results also show a lack of functional toxicity from the CRISPR-Cas9-AAV6 procedure because LT-HSCs were preserved and because normal human hematopoiesis was obtained from the genome-edited cells.

In contrast to Dever et al.^25^, who also used a  CRISPR-Cas9-AAV6 system, in this work we were not simply making a single-nucleotide correction but instead inserting a therapeutic transgene in a precise genomic location while maintaining high targeting efficiencies (median of 45% in CD34^+^ HSPCs and up to 20% in LT-HSCs). This targeted cDNA integration therapeutic approach has the benefit of not only being able to correct >97% of known SCID-X1 pathogenic mutations due to the “universal” strategy design and thus should have broader application as most genetic diseases are caused by mutations throughout the gene.

The safety of the approach is further supported by the lack of karyotypic abnormalities generated in RNP exposed CD34^+^ HSPCs and by INDEL frequencies below the limit of detection using a high-fidelity version of Cas9 at 54 potential off-target sites identified by bioinformatics and cell-based methods. Even using wild-type Cas9, off-target INDELs were only detected at two sites (both at low frequencies (<0.3%)), which were at sites of no known functional significance and did not result in any measurable perturbations in the cell population in all the assays used in this work the most important of which was no evidence of abnormal hematopoiesis in RNP-treated cells. In the course of these studies, we transplanted a full human dose for an infant (the target age that we are planning to treat in a phase I/II clinical trial) into NSG mice (28.4 million CD34^+^ HSPCs), a functional safety standard that the Food and Drug Administration (FDA) has used prior to approving a phase I clinical trial of ZFN editing of CD34^+^ HSPCs^24^. The persistence of *IL2RG* gene corrected cells for 8 months (16 weeks in the primary followed by 16 weeks in the secondary recipient) following transplantation into NSG mice with multi-lineage hematopoiesis and without evidence of abnormal hematopoiesis also highlights the general lack of toxicity of the approach. An important aspect of our studies is that we achieved a median correction frequency of 44.5% without selection in PB patient-derived CD34^+^ HSPCs, a cell source that is being used in lentiviral-based gene therapy trials. These functionally gene corrected CD34^+^ HSPCs showed equivalent engraftment following transplantation into NSG mice as unmanipulated patient-derived CD34^+^ HSPCs, again providing data that the GE manufacturing process was not damaging the cells in a significant way.

We also demonstrated that the “universal” strategy of knocking a codon-optimized wild-type cDNA into the endogenous start site functionally rescues gene function using both in vitro and in vivo assays of T and NK-cell development and function. These results include the rescue of T and NK-cell development and function from patient-derived CD34^+ ^HSPCs. While the ultimate test of the safety and efficacy of our approach will be established during a gene therapy clinical phase I/II trial, we believe that we have shown strong evidence using state-of-the-art, gold standard methods of the safety and efficacy of the  CRISPR-Cas9-AAV6 approach to targeting a cDNA to the endogenous translational start site to functionally correct diseases causing mutations throughout a gene. It is likely, however, that specific details of the cDNA targeting strategy will have to be tailored to each gene in order to achieve the safe and effective levels of expression that are needed.

Our rationale for developing a GE-based gene therapy for SCID-X1 is to provide a safe, efficient, precise, and effective treatment option for patients. Although it is encouraging that improved methods for allo-HSCT are being developed and that the results using lentiviral-based gene therapy for SCID has been shown to be safe and effective, the long-term safety, efficacy, and limitations of these approaches remains to be determined. Thus, it is important to continue develop alternative strategies for curing patients with SCID-X1 using approaches that are less genotoxic (the mutational burden from GE is >1000-fold less than for lentiviral-based modification strategies by comparing the frequency of off-target INDELs to the frequency of uncontrolled lentiviral insertions). Ideally, multiple effective options will be available for patients, their families, and their treating physicians in the future thus giving them the opportunity to choose the approach that best fits their needs and circumstances. In sum, the safety and efficacy data presented in this study provides strong support for the clinical development of functional gene correction using the CRISPR-Cas9-AAV6 GE methodology to establish a long lasting therapeutic, potentially curative, strategy beneficial to >97% of SCID-X1 patients.

## Methods

### CRISPR-Cas9 sgRNA

Seven *IL2RG*, exon 1 specific, 20 nt length oligomer sequences, used in the initial screen, were identified using the online CRISPOR software (crispor.terof.net) and synthesized (Synthego, Redwood City, CA, USA) as part of a chimeric 100 nt sgRNA. Chemically modified sgRNA oligomers were manufactured using a proprietary synthesizer by Synthego Corp. (Redwood City, CA, USA) on controlled-pore glass (AM Chemicals, Carlsbad, CA, USA) using 2′-O-t-butyldimethylsilyl-protected and 2′-O-methyl ribonucleotide amidites (ChemGenes, Wilmington, MA) according to established procedures. Standard ancillary reagents for oxidation, capping and detritylation were used (EMD Millipore, Cincinatti, OH). Formation of internucleotide phosphorothioate linkages was performed using ((dimethylaminomethylidene) amino-3H-1,2,4-dithiazoline-3-thione (DDTT, ChemGenes, Wilmington, MA).

A set of 2′-*O*-methyl 3′phosphorothioate MS[30] modified full-length 20 nt and three additional versions having 1, 2, and 3 nt removed from the 5′ end of the complementary region of the *IL2RG* sgRNA guide #1 were synthesized (TriLink Biotechnologies, San Diego, CA, USA) and purified using reverse phase high-performance liquid chromatography. Purity analysis was confirmed by liquid chromatography–mass spectrometry.

### AAV6-based DNA donor design and vector production

All homology based AAV6 vector plasmids were cloned into pAAV-MCS plasmid containing AAV2-specific inverted terminal repeats (ITRs) (Stratagene now part of Agilent Technologies, Santa Clara, CA, USA) using Gibson Assembly cloning kit according to the instructions in the commercial kit (New England Biolabs, cat # E5510S). Corrective, codon diverged *IL2RG* cDNA was designed to contain silent mutations that generated 78% sequence homology to the endogenous, wild-type gene. All AAV6 viruses were produced in 293T in the presence of 1 ng/ml sodium butyrate (Sigma-Aldrich, cat. no. 303410) cells and purified 48 h later using an iodixanol gradient approach as previously described^5^. The following provides additional detail: all AAV6 viruses were produced in 293T seeded at 14 × 10^6^ cells per dish in 10 15-cm dishes 1 day before transfection. In all, 6 μg ITR-containing plasmid and 22 μg pDGM6 (gift from Dr. David Russell, University of Washington, Seattle, WA, USA), containing the AAV6 cap genes, AAV2 rep genes, and adenovirus helper genes were transfected per one 15-cm dish using PEI at a 4:1 ratio (PEI to DNA). Forty-eight hours post transfection, AAV6 were harvested from cells by three freeze–thaw cycles, followed by a 45-min incubation with TurboNuclease at 250 U/mL (Abnova, Heidelberg, Germany). AAV vectors were purified using Iodixanol density gradient and ultracentrifugation at 48,000 rpms for 2 h at 18 ºC. AAV6 particles were extracted from the 40 to 60% gradient interface and dialyzed, three times, in PBS (phosphate-buffered saline) containing 5% sorbitol. A 10 K MWCO slide-a-lyzer G2 dialysis cassette (Thermo Fisher, Santa Clara, CA, USA) was used for dialyses. Pluronic acid was added to the purified AAV6 at a final concentration of 0.001%, aliquot and stored at −80 °C.

### CD34^+^ HSPCs

Mobilized peripheral blood  (mPB) and bone marow (BM) CD34^+^ HSPCs cells were purchased from AllCells (Alameda, CA, USA). Cells were thawed using published protocol^55^. Freshly purified CB-derived CD34^+^ HSPCs, of male origin, were obtained through the Binns Program for Cord Blood Research at Stanford University, under informed consent. Mononuclear cells (MNCs) isolation was carried out by density gradient centrifugation using Ficoll Paque Plus (400 × *g* for 30 min without brake). Following two platelet washes (200 × *g*, 10–15 min with brake) HSPCs were labeled and positively selected using the CD34^+^ Microbead Kit Ultrapure (Miltenyi Biotec, San Diego, CA, USA) according to manufacturer’s protocol. Enriched cells were stained with Allophycocyanin (APC) anti-human CD34 (clone 561; Biolegend, San Jose, CA, USA) and sample purity was assessed on an Accuri C6 flow cytometer (BD Biosciences, San Jose, CA, USA). Following purification or thawing, CD34^+^ HSCPs were cultured for 36–48 h at 37 °C, 5% CO_2_ and 5% O_2_, at a density of 2.5 × 10^5^ cells/ml in StemSpan SFEM II (Stemcell Technologies, Vancouver, Canada) supplemented with Stem Cell Factor (SCF) (100 ng/ml), Thrombopoietin (TPO) (100 ng/ml), Fms-like tyrosine kinase 3 ligand (Flt3-Ligand) (100 ng/ml), Interleukin 6 (IL-6) (100 ng/ml), StemRegenin 1 (SR1) (0.75 mM), and UM171 (35 nM, Stemcell Technologies).

For secondary engraftment studies, CD34^+^ HSPCs were purified from total BM of NSG mice at end point analysis. Sufficiently pure samples (≥80% CD34^+^) were pooled and cultured at 37 °C, 5% CO_2_, and 5% O_2_ for 12 h prior to secondary transplant.

### T-cell purification

Primary human T cells were obtained from healthy male donors from Stanford University School of Medicine Blood Center after informed consent was obtained and purified by Ficoll density gradient centrifugation followed by red blood cell lysis in ammonium chloride solution (Stemcell Technologies,Vancouver, Canada) and magnetic negative selection using a Pan T-cell isolation kit (Miltenyi Biotec, San Diego, CA, USA) according to manufacturer’s instructions. Cells were cultured at 37 °C, 20% O_2_ and 5% CO_2_ in X-Vivo 15 (Lonza, Walkersville, MD, USA) supplemented with 5% human serum (Sigma-Aldrich, St. Louis, MO, USA) and 100 IU/ml human recombinant IL-2 (Peprotech, Rocky Hill, NJ, USA) and 10 ng/ml human recombinant IL-7 (BD Biosciences, San Jose, CA, USA). Cells were stimulated with immobilized anti-CD3 (OKT3, Tonbo Biosciences, San Diego, CA, USA) and with soluble anti-CD28 (CD28.2, Tonbo Biosciences) for three days prior to electroporation.

### GE and INDEL quantification

Editing of all primary cells was carried out using a ribonucleic protein (RNP) system at a molar ratio of either 1:2.5 or 1:5 (Cas9: sgRNA), unless otherwise stated. Recombinant *S*. *pyogenes* Cas9 protein was purchased from IDT (Integrated DNA Technologies, Coralville, Iowa, USA). Nucleofection was performed in P3 nucleofection solution (Lonza) and Lonza Nucleofector 4d (program DZ-100). Cells were plated at a concentration of 1.0 × 10^5^–2.5 × 10^5^ cells/ml. For T cells editing, electroporation was performed using Lonza Nucleofector 4d (program EO-115) with an RNP composition as used for CD34^+^ HSPCs editing. INDEL frequencies were quantified using TIDE online software on genomic DNA extracted using Quick Extract (Epicentre, an Illumina Company, cat no. QE09050) according to manufacturing specifications.

### Genome targeting and quantification

CD34^+^ HSPCs nucleofected with the *IL2RG*-specific RNP system were plated at a density of 5.0 × 10^5^ cells/ml and transduced with the AAV6 donor at an multiplicity of infection (MOI) of 200,000 vg/μl within 15 min of nucleofection. Cells were cultured at 37 °C, 5% CO_2_, 5% O_2_ for 36 h to 48 h after which they were either re-plated in fresh media, at a density of 2.5 × 10^5^ cells/ml or prepared for xenotransplantation studies.

Absolute quantification of the levels of genomic integration was carried out using Digital Droplet PCR^TM^ (ddPCR^TM^, Bio-Rad, Hercules, CA, USA). Genomic DNA was extracted as described in previous section. In all, 1 μg of genomic DNA was digested with EcoRV-HF (20U) in Cutsmart buffer at 37 °C for 1 h. ddPCR reaction contains 1× reference primer/probe mix synthesized at a 3.6 ratio (900 nM primer and 250 nM FAM labeled probe), 1× target primer/probe mix synthesized at a 3.6 ratio (HEX labeled probe), 1× ddPCR Supermix for probe without dUTP, 50 ng of digested DNA and water for a total volume of 25 μl. The primers and probes sequences are detailed in Table S2.

Genomic DNA in the ddPCR mixture was partitioned into individual droplets using QX100 Droplet Generator, transferred to a 96-deep well PCR plate and amplified in a Bio-Rad PCR thermocycler. The following ddPCR program was optimized to amplify a 500-bp amplicon: step 1—95 °C for 10 min, ramp 1 °C/s, step 2—94 °C for 30 s, ramp 1 °C/s, step 3—60.8 °C for 30 s, ramp 1 °C/s, step 4—72 °C for 2 min, ramp 1 °C/s, step 5—repeat steps 2–4 for 50 cycles, step 6—98 °C for 10 min, ramp 1 °C/s, step 7—4 °C, ramp 1 °C/s. Bio-Rad Droplet Reader and QuantaSoft Software were used to read and analyzed the experiment following manufacturer’s guidelines (Bio-Rad). Absolute quantification as copy of DNA/μl was determined for the reference, endogenous *IL2RG* gene and for the integrated *IL2RG* cDNA. Percent targeting in total population was calculated as a ratio of HEX to FAM signal. For all targeting experiments, genomic DNA was derived from male donors.

Quantification of *IL2RG* cDNA targeted integration frequencies in SCID-Xl patients was assessed based on agarose gel quantification as *IL2RG* cDNA signal ratio intensity.

### Methylcellulose CFU assay

Two days post genome targeting, single cells were sorted onto 96-well plates coated with MethoCult Optimum (StemCell Technologies, cat no H4034). Fourteen days later, colonies derived from targeted and mock-treated cells were counted and scored based on morphological features pertaining to Colony Forming Units-erytroid (CFU-E), erythroid burst forming units (BFU-E), Colony Forminig Unit- Granulocytes, Monocytes (CFU-GM), and CFU-GEMM. Genotyping analysis was performed to quantify the percent of mono-allelic targeting. A three primer-based *IL2RG*-specific genotyping PCR-based protocol was established an optimized as follows: *IL2RG* WT-F1 5′-GGGTGACCAAGTCAAGGAAG-3′; int-*IL2RG*-R1: 5′-GATGGTGGTATTCAAGCCGACCCCGA-3′; *IL2RG* WT-R2: 5′-AATGTCCCACAGTATCCCTGG-3′. The PCR reaction contained 0.5 μM of each of the three primer, 1× Phusion Master Mix High Fidelity, 150−200 ng of genomic DNA and water to a final volume of 25 μl. The following PCR program generated an integration band of 543 bp from F1 and R1 primer set and an endogenous band of 1502 bp from F1 and R2 primer set: step 1—98 °C for 30 s, step 2—98 °C for 10 s; step 3—66 °C for 30 s; step 4—72 °C for 30 s, step 5—repeat steps 2–4 for a total of 30 cycles, step 6—72 °C for 7 min; step 7—4 °C.

### OP9-idll1 system

OP9 cells were generously provided by Dr. Irving Weissman’s lab and generated as previously described^40^. Briefly, OP9 stromal cells were infected with two lentiviral constructs, the first containing a TET-ON tetracycline trans-activator (rtTA3) under control of a constitutive promoter (EF1a) and linked to turboRFP, and the second containing the Dll1 gene under control of a tet-responsive element (TRE) promoter and linked to turboRFP. In the presence of tetracycline or doxycyline, the rtTA3 rapidly activates expression of Dll1 and turboRFP.

### Lymphoid differentiation of patient-derived CD34^+^ HSPCs

SCID-X1 patient-derived CD34^+^ HSPCs were targeted with the *IL2RG* cDNA corrective donor. Forty-eight hours post targeting, 300 cells derived from either un-target or *IL2RG* cDNA targeted were sorted onto a well of a 96-well plate seeded with 50,000 OP9-idll1 cells 48 h in advance. Cells were incubated at 37 °C, 5% CO_2_, 10% O_2_ for 1 week in activation media containing: alpha-MEM base media (ThermoFisher, cat no. 32561102), supplied with 10% fetal bovine serum (FBS; GemCell, cat no. 100-500), mono-thioglycerol (MTG) (100 μM), ascorbic acid (50 μg/ml), 1× penicillin/streptomycin, SCF (10 ng/ml, PeproTech, cat no. AF-300-07), Flt-3L (5 ng/ml, PeproTech, cat no. AF-300-19), IL-7 (5 ng/ml PeproTech, cat no. 200-07), IL-3 (3.3 ng/ml, PeproTech cat no. AF-200-03), Granulocyte-macrophage colony-stimulating factor (10 ng/ml, PeproTech, cat no. AF-300-03), TPO (10 ng/ml, PeproTech cat no. AF-300-18), EPO (2 U/ml, PeproTech, cat no. 100-64), IL-15 (10 ng/ml, PeproTech cat no. AF-200-15), IL-6 (10 ng/ml, PeproTech, cat no. 200-06). After 7 days, half the medium was exchanged and DOX was added at a final concentration of 1 μg/ml.

### In vitro multi-lineage differentiation analysis

Lymphoid, myeloid, and erythroid differentiation potential was determined using FACS analysis at 1 week post DOX induction. In all, 100% growth was obtained from all wells seeded with 300 targeted or mock-treated cells. Media were removed from all positive wells and cells were washed in 1× PBS. Cells were re-suspended in 50 μl MACS buffer (1× PBS, 2% FBS, 2 mM EDTA), blocked for nonspecific binding (5% vol/vol human FcR blocking reagent, Miltenyi, cat no. 130-059-901), stained for live dead discrimination using Live/Dead blue dead cell staining kit for UV (ThermoFisher Scientific, cat no. L23105) and stained (30 min, 4 °C dark) using CD3 PerCP/Cy5.5 (HiT3A, BioLegend), CD4 BV650 (OKT4, BioLegend), CD8 APC (HiT8a, BioLegend), CD11c BV605 (3.9, BioLegend), CD14 BV510 (M5E2, BioLegend), CD19 FITC (HIB19, BioLegend), CD33 AF-300 (WM53, BD Pharmingen), CD45 BV786 (BD Pharmingen), CD56 PE (MEM-188 BioLegend), CD235a PE-Cy7 (HI264, BioLegend), and CD271 (tNGFR) CF-594 (C40-1457, BD Horizon).

### Phosphorylated STAT5 in vitro assay

To assess STAT5 phosphorylation in response to cytokine stimulation, purified human T cells were cultured for 7 days post electroporation and starved, overnight, in medium lacking serum and cytokines. Samples were split and either stimulated with IL-2 (100 U/ml) and IL-7 (10 ng/ml) or left unstimulated. Cells were split again, fixed, permeabilized using 4% PFA and methanol and stained with CD3 PE (UCHT1, BioLegend), CD271 (tNGFR) APC (ME20.4, Biolegend). Intracellular antigens were stained with pSTAT5 AF-488 (pY694, BD Bioscience) or isotype control (BD Biosciences). FACS analysis was performed on Accuri C6 (BD Biosciences) or Cytoflex (Beckman Coulter) and data analysis was performed using FlowJo.

### CFSE cellular proliferation of *IL2RG* targeted human T cells

Purified human T cells were nucleofected alone (mock treated) or in the presence of the long corrective *IL2RG* cDNA-tNGFR DNA donor vector. NGFR^bright^ T cells were sorted. NGFR^bright^ or mock-treated cells were labeled with CFSE (BioLegend) according to the manufacturer’s protocol and either re-stimulated with anti-CD3/anti-CD28/IL-2/IL-7 as described in previous section or left unstimulated (IL-7 only). Targeting levels were monitored and quantified based on the tNGFR expression and on absolute quantification of the integrated *IL2RG* cDNA by ddPCR.

### Xenotransplantation of genome targeted CD34^+^ HSPCs into mice

For all human engraftment studies, we used freshly purified CB derived CD34^+^ HSPCs derived from healthy male donors, under informed consent. Human engraftment studies designed to rescue the disease phenotype were carried out using frozen, mPB CD34^+^ HSPCs derived from SCID-Xl patients 1–3. SCID-X1 patients were given subcutaneous injections of Granulocytes Colony-Stimulating Factor (G-CSF) (filgrastim, Neupogen®; Amgen, Thousand Oaks, CA) for 5 consecutive days at 10–16 mcg/kg/day and one dose of Pleraxifor for mobilization and apheresis (National Institutes of Allergy and Infectious Disease IRB-approved protocol 94-I-0073). PB CD34^+^ HSPCs were selected from the leukepheresis product using Miltenyi CliniMACS.

Human engraftment experimental design and mouse handling followed an approved Stanford University Administrative Panel on Lab Animal Care (APLAC). Cells used for engraftment studies were exposed to a maximum of 4 days ex vivo culturing.

### IH primary (1°) human engraftment

In all, 1.0 × 10^5^ to 2.5 × 10^5^ cells derived from *IL2RG* cDNA targeted cells or mock-treated cells (electroporated in the absence of RNP and never exposed to AAV6) were re-suspended in 25−30 μl of freshly prepared CD34^+^ complete media with the addition of UM171 and SR1.

Three to 4 days old NSG pups were irradiated with 100 cGy and immediately engrafted IH using an insulin syringe with a 27 gauge × ½” needle. A total of 2.15 × 10^6^ cells from each condition were injected into 11 pups/condition. In all, 18/22 engrafted pups were analyzed at week 16 post engraftment.

Level of human engraftment was assessed at weeks 8 and 12 using BM aspirates and PB samples. At week 16 or later, end point analysis was done from total BM, SP, liver, and PB. For total BM analysis, mouse bones were harvested from tibiae, femurs, sternum, and spinal cord from each mouse and grinded using a mortar and pestle. MNCs were purified using Ficoll gradient centrifugation (Ficoll-Paque Plus, GE Healthcare, Sunnyvale, CA, USA) for 25 min at 2000 × *g*, at room temperature. SP and liver samples were grinded against a 40 μM mesh, transferred to a FACS tube and spun down at 300 × *g* for 5 min, at 4 °C. Red blood cells were lysed following a 10- to 12-min incubation on ice with 500 μl of 1× ACK lysis buffer (ThermoScientific, cat no. A1049201). Reaction was quenched and cells were washed with MACS buffer (2–5% FBS, 2 mM EDTA, and 1× PBS). PB samples were treated with 500 μl of 2% Dextren and incubated at 37 C for 30 min to 1 h. In all, 800 μl to 1 ml of the top layer was transferred to a FACS tube, spun down at 300 × *g*, 5 min and red blood cells lysed as already described.

Cells purified from all four sources were re-suspended in 50 μl MACS buffer, blocked, stained with LIVE/Dead staining solution and stained for 30 min at 4 °C, dark with the following antibody panel: CD3 PerCP/Cy5.5 (HiT3A, BioLegend), CD19 FITC (HIB19, BioLegend), mCD45.1 PE-Cy7 (A20, BioLegend), CD16 PE-Cy5 (3G8, BD Pharmingen), CD235a PE (HI264, BioLegend), HLA A-B-C APC-Cy7 (W6/32, BioLegend), CD33 AF-300 (WM53, BD Pharmingen), CD8 APC (HiT8a, BioLegend), CD45 BV786 (HI3a, BD Horizon), CD4 BV650 (OKT4, BioLegend), CD11c BV605 (BioLegend), CD14 BV510 (M5E2, BioLegend), and CD56 Pacific Blue (MEM-188, BioLegend).

### IF primary (1°) human engraftment

In all, 5.0 × 10^5^ cells derived from WT cells, mock treated, RNP treated, and *IL2RG* cDNA targeted cells were injected IF into 6–8 weeks old NSG mice. Mice were irradiated with 200 cGy 2–4 h prior to engraftment. Cells were prepared in the same fashion as described in the IH section. A total of 2.0 × 10^6^ WT cells were injected into a total of four mice, 3.5 × 10^6^ mock-treated cells were injected into seven mice, 2.0 × 10^6^ RNP-treated cells were injected into four mice and 7.5 × 10^6^ *IL2RG* cDNA targeted cells were injected into 15 mice. In all, 29/30 injected mice were analyzed at week 16 post engraftment, as described in the IH engraftment assay section.

### Secondary (2°) human engraftment

Secondary engraftments experiments were derived from both IH and from IF engrafted human cells. From the IH mock and *IL2RG* cDNA targeted engrafted mice, total BM was collected at week 16 post primary engraftment, MNC were purified using Ficoll gradient centrifugation and CD34^+^ cells were enriched using CD34^+^ microbeads (Miltenyi). Enriched cells were pooled from five engrafted mice with mock-treated cells and from seven engrafted mice with *IL2RG* cDNA targeted cells and cultured overnight in complete CD34^+^ media containing UM171 and SR1. Following overnight incubation, cellular count and viability was determined for mock-treated cells to be 2.47 × 10^6^ cells at 85.5% viability and for *IL2RG* cDNA targeted cells was 4.8 × 10^6^ cells at 84% viability. In all, 3.5 × 10^5^ mock-treated cells and 5.0 × 10^5^ *IL2RG* cDNA targeted cells were engrafted IF into eight 6–8 weeks old, irradiated NSG mice (four males and four females).

Secondary engraftment experiments derived from IF primary engraftments were carried on as described above with the following modification: 5.0 × 10^5^ CD34^+^ enriched cells derived from WT, mock and RNP primary engraftment assay were IF injected into four 6–8 weeks old NSG mice, 5.0 × 10^5^ CD34^+^ enriched cells derived from *IL2RG* cDNA targeted cells were IF injected into 12 6–8 weeks old NSG mice. Equal numbers of male and female mice were used.

### IH primary (1°) human engraftment

Frozen mPB CD34^+^ HSPCs derived from SCID-Xl patients were thawed and genome targeted as described in previous section. In all, 2.5 × 10^5^ cells were IH injected into 3–4 days old, irradiated NSG pups.

### GUIDE-Seq

sgRNAs were generated by cloning annealed oligos containing the IL2RG target sequence into pX330 (Gift from Feng Zhang, Addgene #42230)^56^. In all, 200,000 U2OS cells (ATCC #HTB-96) were nucleofected with 1 μg of pX330 Cas9 and gRNA plasmid and 100 pmol dsODN using SE cell line nucleofection solution and the CA-138 program on a Lonza 4D-nucleofector. The nucleofected cells were seeded in 500 μl of McCoy’s 5a Medium Modified (ATCC) in a 24-well plate. Genomic DNA (gDNA) was extracted 3 days post nucleofection using a Quick-DNA Miniprep plus kit (Zymo Research). Successful integration of the dsODN was confirmed by RFLP assay with NdeI. In all, 400 ng of gDNA was sheared using a Covaris LE220 Ultrasonicator to an average length of 500 bp. Samples were prepared for Guide-seq^41^ and sequenced on the Illumina Miseq. Briefly, solid-phase reversible immobilization magnetic beads were used to isolate genomic DNA, which was further sheared to an averaged of 500 bp (Covaris S200), end-repaired and ligated to adaptors containing 8-nt random molecular index. Target enrichment was achieved through two rounds of nested PCR using primers complementary to the oligo tag. We analyzed GUIDE-Seq data using the standard pipeline^41^ with a reduced gap penalty for better detection of off-target sites containing DNA or RNA bulges.

### Bioinformatic off-target identification

Potential off-target sites for the IL2RG gRNA in the human genome (hg19) were identified using the web tool COSMID^42^ with up to three mismatches allowed in the 19 PAM (protospacer adjacent motif) proximal bases. After off-target site ranking, 45 sites were selected for off-target screening.

### Off-target validation

Frozen mPB CD34^+^ cells (AllCells) were electroporated with 300 μg/ml of Cas9 and 160 μg/ml of sgRNA. sgDNA was extracted 48 h after RNP delivery. Off-target sites were amplified by locus-specific PCR. PCR primers contained adapter sequences to facilitate amplicon barcoding via a second round of PCR as previously described^57^. All amplicons were pooled at an equimolar ratio and sequenced on the Illumina Miseq according to manufacturer’s instructions using custom sequencing primers for Read 2 and Read Index. Sequencing data were analyzed using a custom INDEL quantification pipeline^58^.

### Karyotype analysis

Fresh CB CD34^+^ HSPCs were purified, genome edited or targeted as previously described. Four days post ex vivo culturing and manipulations, 5 × 10^5^ cells from WT untreated, mock, RNP only, RNP and AAV6 or AAV6 only treated cells were processed by Stanford Cytology Labs at Stanford University. Karyotyping analysis was performed on 20 cells derived from each condition.

### IL2RG-specific genotoxicity assays in human cell lines

Levels of γH2AX induced by different classes of engineered nucleases were quantified by measuring the phosphorylation of histone H2AX, a marker of DSB formation. K562 cells were nucleofected with the indicated doses of each nuclease expression plasmid, and the percentage of γH2AX^+^ cells was measured by FACS at 48 h post nucleofection.

293T cells were co-transfected with plasmids expressing GFP and nuclease. GFP-positive cells were analyzed at day 2 and again at day 6 by FACS. Percent survival relative to I-SceI control was calculated as follows:

$$\frac{{{\mathrm{Nuclease}}\;{\mathrm{day}}\;6/{\mathrm{Nuclease}}\;{\mathrm{day}}\;2}}{{{\mathrm{I}}-{\mathrm{SceI}}\;{\mathrm{day}}\;6/{\mathrm{I-SceI}}\;{\mathrm{day}}\;2}} \times 100$$

A percent equal to 100 denotes no toxicity while a percentage <100 marks toxicity.

### FACS analysis

All FACS analysis pertaining to OP9-idll1 and human engraftment analysis were done on FACS Aria II SORT instrument part of FACS Facility Core from Stanford University, Institute for Stem Cell Biology and Regenerative Medicine.

### Statistical analysis

Statistical analysis was done with Prism 7 (GraphPad Software).

### Ethics and animal approval statement

All of the studies performed in this work comply with all relevant ethical regulations. The animal studies were reviewed, approved, and monitored by the Stanford University IACUC.

### Reporting summary

Further information on experimental design is available in the Nature Research Reporting Summary linked to this article.

## Supplementary information

Supplementary Information

Reporting Summary

## Data Availability

Sequencing data have been deposited at [BioProject ID PRJNA526905] under accession code PRJNA526905 [http://www.ncbi.nlm.nih.gov/bioproject/526905]. The authors declare that the data supporting the findings of this study are available within the paper and its supplementary information files or from the authors upon reasonable request.

## source data

source Data

## References

[1] Pai SY (2014). Transplantation outcomes for severe combined immunodeficiency, 2000-2009. N. Engl. J. Med..

[2] De Ravin SS (2016). Lentiviral hematopoietic stem cell gene therapy for X-linked severe combined immunodeficiency. Sci. Transl. Med..

[3] Stephan V (1996). Atypical X-linked severe combined immunodeficiency due to possible spontaneous reversion of the genetic defect in T cells. N. Engl. J. Med..

[4] Hacein-Bey-Abina S (2003). LMO2-associated clonal T cell proliferation in two patients after gene therapy for SCID-X1. Science.

[5] Woods NB, Bottero V, Schmidt M, von Kalle C, Verma IM (2006). Gene therapy: therapeutic gene causing lymphoma. Nature.

[6] Hacein-Bey-Abina S (2008). Insertional oncogenesis in 4 patients after retrovirus-mediated gene therapy of SCID-X1. J. Clin. Invest..

[7] Six, E., et al. in *ASGCT 20*^*th*^ Meeting, Washington DC, Abstract #753 (2017).

[8] Wu C, Dunbar CE (2011). Stem cell gene therapy: the risks of insertional mutagenesis and approaches to minimize genotoxicity. Front. Med..

[9] Aiuti Alessandro, Roncarolo Maria Grazia (2009). Ten years of gene therapy for primary immune deficiencies. Hematology.

[10] Fischer A, Hacein-Bey-Abina S, Cavazzana-Calvo M (2010). 20 years of gene therapy for SCID. Nat. Immunol..

[11] Smogorzewska EM, Weinberg KI, Kohn DB (2003). [Transplantation of genetically modified cells in the treatment of children with SCID: great hopes and recent disappointments]. Med. Wieku. Rozwoj..

[12] Ott MG (2006). Correction of X-linked chronic granulomatous disease by gene therapy, augmented by insertional activation of MDS1-EVI1, PRDM16 or SETBP1. Nat. Med..

[13] Kang EM (2010). Retrovirus gene therapy for X-linked chronic granulomatous disease can achieve stable long-term correction of oxidase activity in peripheral blood neutrophils. Blood.

[14] Boztug K (2010). Stem-cell gene therapy for the Wiskott-Aldrich syndrome. N. Engl. J. Med..

[15] Cavazzana M, Six E, Lagresle-Peyrou C, Andre-Schmutz I, Hacein-Bey-Abina S (2016). Gene therapy for X-linked severe combined immunodeficiency: where do we stand?. Hum. Gene Ther..

[16] Hacein-Bey-Abina S (2014). A modified gamma-retrovirus vector for X-linked severe combined immunodeficiency. N. Engl. J. Med..

[17] Thornhill SI (2008). Self-inactivating gammaretroviral vectors for gene therapy of X-linked severe combined immunodeficiency. Mol. Ther..

[18] Voit RA, Hendel A, Pruett-Miller SM, Porteus MH (2014). Nuclease-mediated gene editing by homologous recombination of the human globin locus. Nucleic Acids Res..

[19] Schiroli Giulia, Ferrari Samuele, Conway Anthony, Jacob Aurelien, Capo Valentina, Albano Luisa, Plati Tiziana, Castiello Maria C., Sanvito Francesca, Gennery Andrew R., Bovolenta Chiara, Palchaudhuri Rahul, Scadden David T., Holmes Michael C., Villa Anna, Sitia Giovanni, Lombardo Angelo, Genovese Pietro, Naldini Luigi (2017). Preclinical modeling highlights the therapeutic potential of hematopoietic stem cell gene editing for correction of SCID-X1. Science Translational Medicine.

[20] Genovese P (2014). Targeted genome editing in human repopulating haematopoietic stem cells. Nature.

[21] Porteus MH, Baltimore D (2003). Chimeric nucleases stimulate gene targeting in human cells. Science.

[22] Porteus MH, Cathomen T, Weitzman MD, Baltimore D (2003). Efficient gene targeting mediated by adeno-associated virus and DNA double-strand breaks. Mol. Cell. Biol..

[23] Shalem O (2014). Genome-scale CRISPR-Cas9 knockout screening in human cells. Science.

[24] DiGiusto DL (2016). Preclinical development and qualification of ZFN-mediated CCR5 disruption in human hematopoietic stem/progenitor cells. Mol. Ther. Methods Clin. Dev..

[25] Dever DP (2016). CRISPR/Cas9 beta-globin gene targeting in human haematopoietic stem cells. Nature.

[26] Urnov FD (2005). Highly efficient endogenous human gene correction using designed zinc-finger nucleases. Nature.

[27] Connelly JP, Barker JC, Pruett-Miller S, Porteus MH (2010). Gene correction by homologous recombination with zinc finger nucleases in primary cells from a mouse model of a generic recessive genetic disease. Mol. Ther..

[28] Li T (2011). TAL nucleases (TALNs): hybrid proteins composed of TAL effectors and FokI DNA-cleavage domain. Nucleic Acids Res..

[29] Jinek M (2012). A programmable dual-RNA-guided DNA endonuclease in adaptive bacterial immunity. Science.

[30] Hendel A (2015). Chemically modified guide RNAs enhance CRISPR-Cas genome editing in human primary cells. Nat. Biotechnol..

[31] De Ravin Suk See, Li Linhong, Wu Xiaolin, Choi Uimook, Allen Cornell, Koontz Sherry, Lee Janet, Theobald-Whiting Narda, Chu Jessica, Garofalo Mary, Sweeney Colin, Kardava Lela, Moir Susan, Viley Angelia, Natarajan Pachai, Su Ling, Kuhns Douglas, Zarember Kol A., Peshwa Madhusudan V., Malech Harry L. (2017). CRISPR-Cas9 gene repair of hematopoietic stem cells from patients with X-linked chronic granulomatous disease. Science Translational Medicine.

[32] Eyquem J (2017). Targeting a CAR to the TRAC locus with CRISPR/Cas9 enhances tumour rejection. Nature.

[33] Fu Y, Sander JD, Reyon D, Cascio VM, Joung JK (2014). Improving CRISPR-Cas nuclease specificity using truncated guide RNAs. Nat. Biotechnol..

[34] Sentmanat MF, Peters ST, Florian CP, Connelly JP, Pruett-Miller SM (2018). A survey of validation strategies for CRISPR-Cas9 editing. Sci. Rep..

[35] Inlay MA (2014). Identification of multipotent progenitors that emerge prior to hematopoietic stem cells in embryonic development. Stem Cell. Rep..

[36] Hong C, Luckey MA, Park JH (2012). Intrathymic IL-7: the where, when, and why of IL-7 signaling during T cell development. Semin. Immunol..

[37] Seet CS (2017). Generation of mature T cells from human hematopoietic stem and progenitor cells in artificial thymic organoids. Nat. Methods.

[38] Lu PH, Negrin RS (1994). A novel population of expanded human CD3+CD56+cells derived from T cells with potent in vivo antitumor activity in mice with severe combined immunodeficiency. J. Immunol..

[39] Traggiai E (2004). Development of a human adaptive immune system in cord blood cell-transplanted mice. Science.

[40] Orr SJ (2010). Implications for gene therapy-limiting expression of IL-2R gamma c delineate differences in signaling thresholds required for lymphocyte development and maintenance. J. Immunol..

[41] Tsai SQ (2015). GUIDE-seq enables genome-wide profiling of off-target cleavage by CRISPR-Cas nucleases. Nat. Biotechnol..

[42] Cradick TJ, Qiu P, Lee CM, Fine EJ, Bao G (2014). COSMID: a web-based tool for identifying and validating CRISPR/Cas off-target sites. Mol. Ther. Nucleic Acids.

[43] Vakulskas CA (2018). A high-fidelity Cas9 mutant delivered as a ribonucleoprotein complex enables efficient gene editing in human hematopoietic stem and progenitor cells. Nat. Med..

[44] Bak, R. O. et al. Multiplexed genetic engineering of human hematopoietic stem and progenitor cells using CRISPR/Cas9 and AAV6. *Elife*10.7554/eLife.27873 (2017). 10.7554/eLife.27873 PMC5656432 28956530

[45] Perez EE (2008). Establishment of HIV-1 resistance in CD4+T cells by genome editing using zinc-finger nucleases. Nat. Biotechnol..

[46] Tebas P (2014). Gene editing of CCR5 in autologous CD4 T cells of persons infected with HIV. N. Engl. J. Med..

[47] Holt N (2010). Human hematopoietic stem/progenitor cells modified by zinc-finger nucleases targeted to CCR5 control HIV-1 in vivo. Nat. Biotechnol..

[48] Hendel A (2014). Quantifying genome-editing outcomes at endogenous loci with SMRT sequencing. Cell Rep..

[49] Pruett-Miller SM, Reading DW, Porter SN, Porteus MH (2009). Attenuation of zinc finger nuclease toxicity by small-molecule regulation of protein levels. PLoS. Genet..

[50] Menon T (2015). Lymphoid regeneration from gene-corrected SCID-X1 subject-derived iPSCs. Cell. Stem. Cell..

[51] Matsubara Y (2014). Transcription activator-like effector nuclease-mediated transduction of exogenous gene into IL2RG locus. Sci. Rep..

[52] Hoban MD (2015). Correction of the sickle cell disease mutation in human hematopoietic stem/progenitor cells. Blood.

[53] Ishikawa F (2005). Development of functional human blood and immune systems in NOD/SCID/IL2 receptor {gamma} chain(null) mice. Blood.

[54] McDermott SP, Eppert K, Lechman ER, Doedens M, Dick JE (2010). Comparison of human cord blood engraftment between immunocompromised mouse strains. Blood.

[55] Denning SM, Tuck DT, Singer KH, Haynes BF (1987). Human thymic epithelial cells function as accessory cells for autologous mature thymocyte activation. J. Immunol..

[56] Cong L (2013). Multiplex genome engineering using CRISPR/Cas systems. Science.

[57] Lee CM, Cradick TJ, Bao G (2016). The Neisseria meningitidis CRISPR-Cas9 system enables specific genome editing in mammalian cells. Mol. Ther..

[58] Lin Y (2014). CRISPR/Cas9 systems have off-target activity with insertions or deletions between target DNA and guide RNA sequences. Nucleic Acids Res..

