# Author Correction: Gene correction for SCID-X1 in long-term hematopoietic stem cells

**DOI:** 10.1038/s41467-019-13620-5

**Published:** 2019-12-04

**Authors:** Mara Pavel-Dinu, Volker Wiebking, Beruh T. Dejene, Waracharee Srifa, Sruthi Mantri, Carmencita E. Nicolas, Ciaran Lee, Gang Bao, Eric J. Kildebeck, Niraj Punjya, Camille Sindhu, Matthew A. Inlay, Nivedita Saxena, Suk See DeRavin, Harry Malech, Maria Grazia Roncarolo, Kenneth I. Weinberg, Matthew H. Porteus

**Affiliations:** 1 Department of Pediatrics, Division of Stem Cell Transplantation and Regenerative Medicine, Stanford University, Stanford, CA 94305 USA; 2 Department of Bioengineering, Rice University, Houston, TX 77030 USA; 3 Center for Engineering Innovation, University of Texas at Dallas, Richardson, TX 75080 USA; 4 Department of Cellular and Molecular Biosciences, University of California Irvine, Irvine, CA 92697 USA; 5 Laboratory of Host Defenses, National Institutes of Allergy and Infectious Diseases, National Institute of Health, Bethesda, MD 20892 USA; 6 Present Address: University of California Davis, School of Medicine, Sacramento, CA 95817 USA

**Keywords:** Genetic engineering, CRISPR-Cas9 genome editing, Haematopoietic stem cells

Correction to: *Nature Communications* 10.1038/s41467-019-09614-y, published online 09 April 2019.

The original version of this Article omitted the following from the last sentence of the Acknowledgements:

This work was supported through an NIH Bench-to-Bedside award made possible by the Office of Rare Diseases Research and NIH Bench-to-Bedside Program funds. This has now been corrected in both the PDF and HTML versions of the Article.

