# Supplementary material for: Gene correction for SCID-X1 in long-term hematopoietic stem cells
Source: Nat Commun. 2019 Apr 9;10:1634. doi: 10.1038/s41467-019-09614-y (PMC6456568; doi:10.1038/s41467-019-09614-y)
Supplement: Supplementary file 1 — Supplementary Information [file 41467_2019_9614_MOESM1_ESM.pdf]

Supplementary Figures

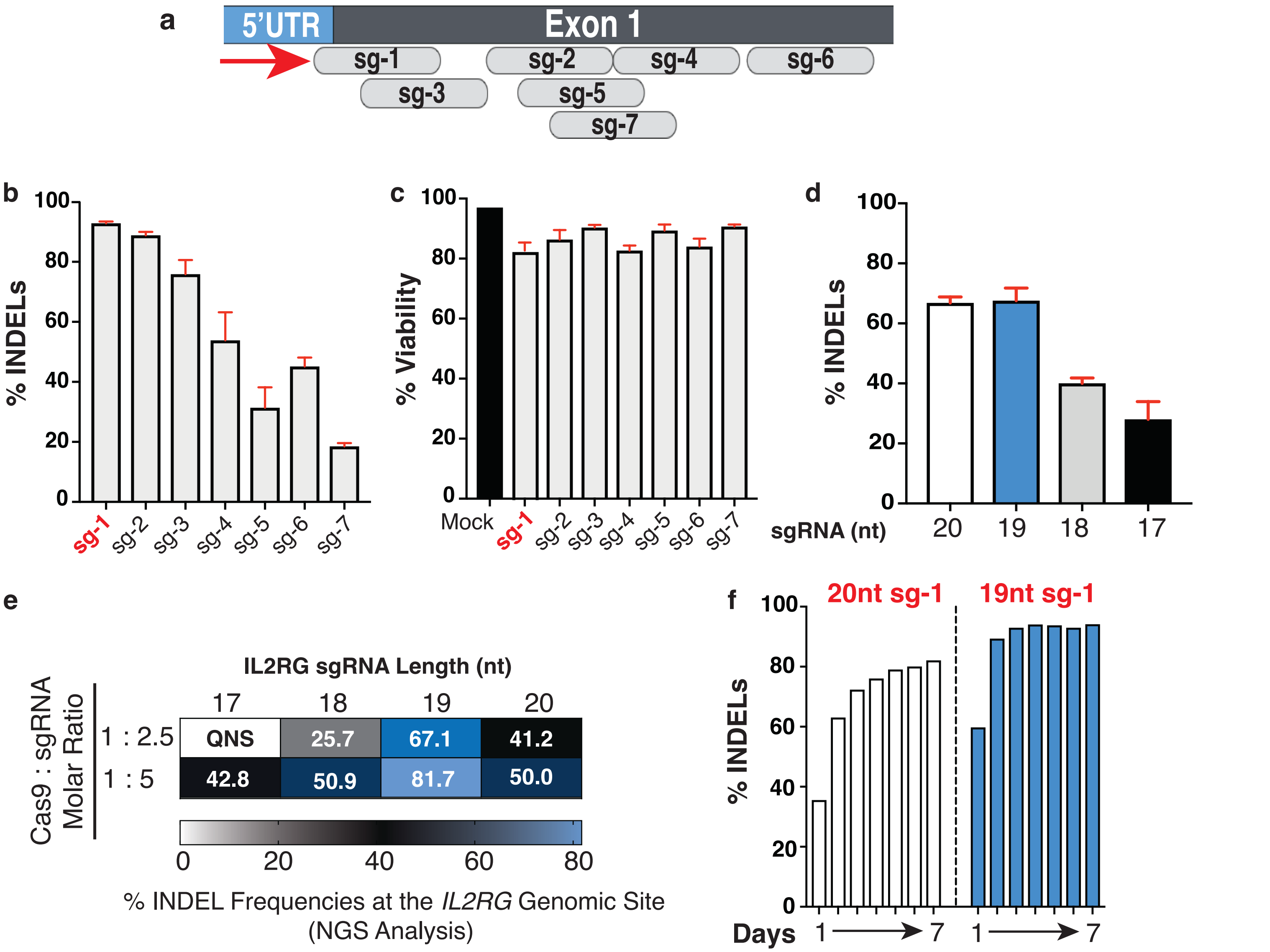

Supplementary Figure 1. Screening and characterization of IL2RG sgRNA guides. (**a**) Schematic of IL2RG sgRNAs for exon 1. (**b**) Percent INDELs and (**c**) Percent viability at day 4 for IL2RG sgRNAs 1-7 nucleofected as RNP in UCB derived CD34^{+} HSPCs (n =1). (**d**) Comparing percent INDELs of WT (20nt) sg-1 IL2RG sgRNA to truncated versions (19nt, 18nt and 17nt) at 1:2.5 molar ratio (n = 2). (**e**) Next generation sequencing (NGS) analysis of samples from **d** (n =1). (**f**) Time course of percent INDELs generated by WT sg-1 IL2RG sgRNA (white bars) and 19nt truncated IL2RG sgRNA (blue bars) (shown n=1). UCB, umbilical cord blood. Mean ± s.e.m

Supplementary Figures

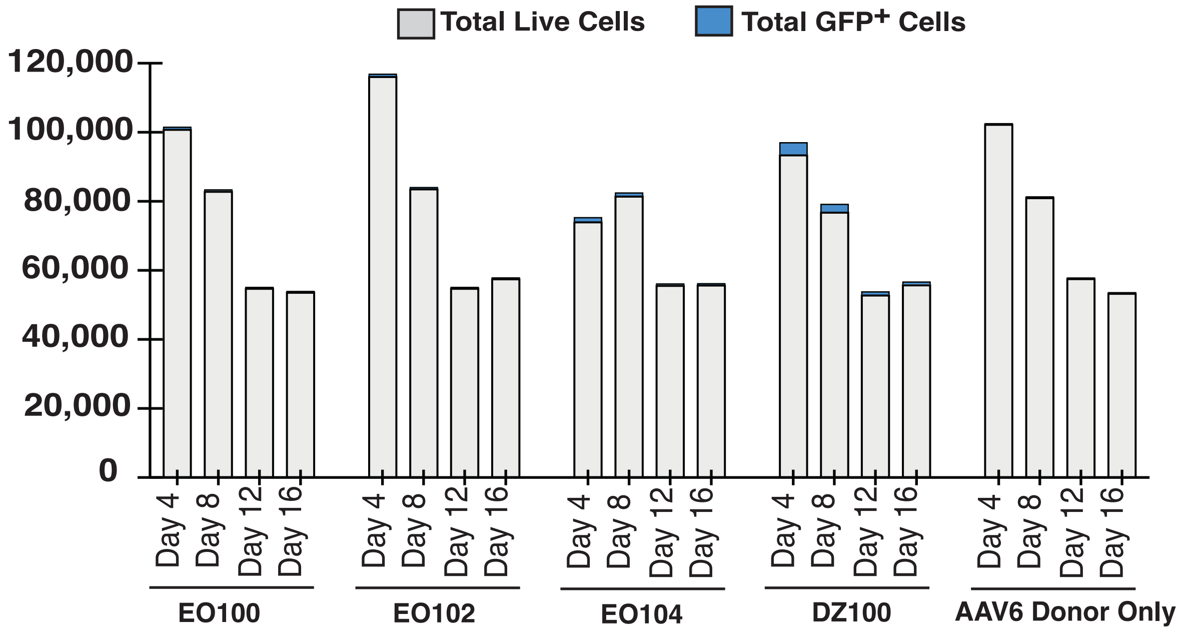

Nucleofection Programs	EO100		EO102		EO104		DZ100		AAV6 Donor Only	
Days Post Nucleofection	Total Live Cells	Total GFP+ Cells	Total Live Cells	Total GFP+ Cells	Total Live Cells	Total GFP+ Cells	Total Live Cells	Total GFP+ Cells	Total Live Cells	Total GFP+ Cells
Day 4	101,001	765	116,300	839	74,231	1,368	93,616	3,664	102,478	65
Day 8	83,050	528	83,755	488	81,685	1,034	77,028	2,377	81,245	119
Day 12	54,950	236	54,982	215	55,818	491	53,003	1,062	57,762	65
Day 16	53,801	158	57,730	186	55,935	446	55,935	938	53,512	54

Supplementary Figure 2. Screening Lonza 4d nucleofection programs in human $CD34^+$ HSPCs. $1x10^5$ cord blood derived $CD34^+$ HSPCs nucleofected with RNP at 1: 2.5 molar ratio and transduced with AAV6 donor DNA virus for CCR5 locus at an MOI of 100,000, as determined by q-PCR. Total live cells as determined by trypan blue staining and % GFP^+ cells by flow cytometry (FACS).

Supplementary Figures

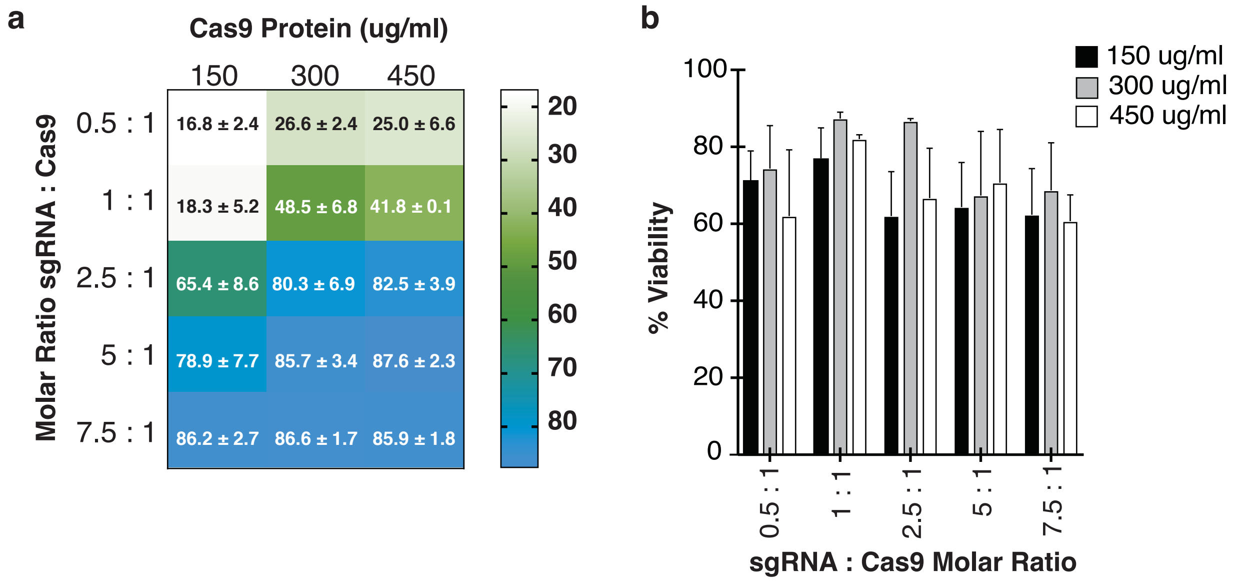

Supplementary Figure 3. IL2RG sgRNA: WT Cas9 protein (RNP) molar ratios in mobilized PB CD34+ HSPCs. (**a**) Heat map of TIDE analysis of various RNP molar ratios as determined at day 4 post genome editing. Shown is percent on target INDELs (n= 3, biological replicates). (**b**) Cellular viability at day 4-post nucleofection of various molar ratio RNPs. Measurement is based on trypan blue staining. Mean ± s.e.m; PB, peripheral blood.

Supplementary Figures

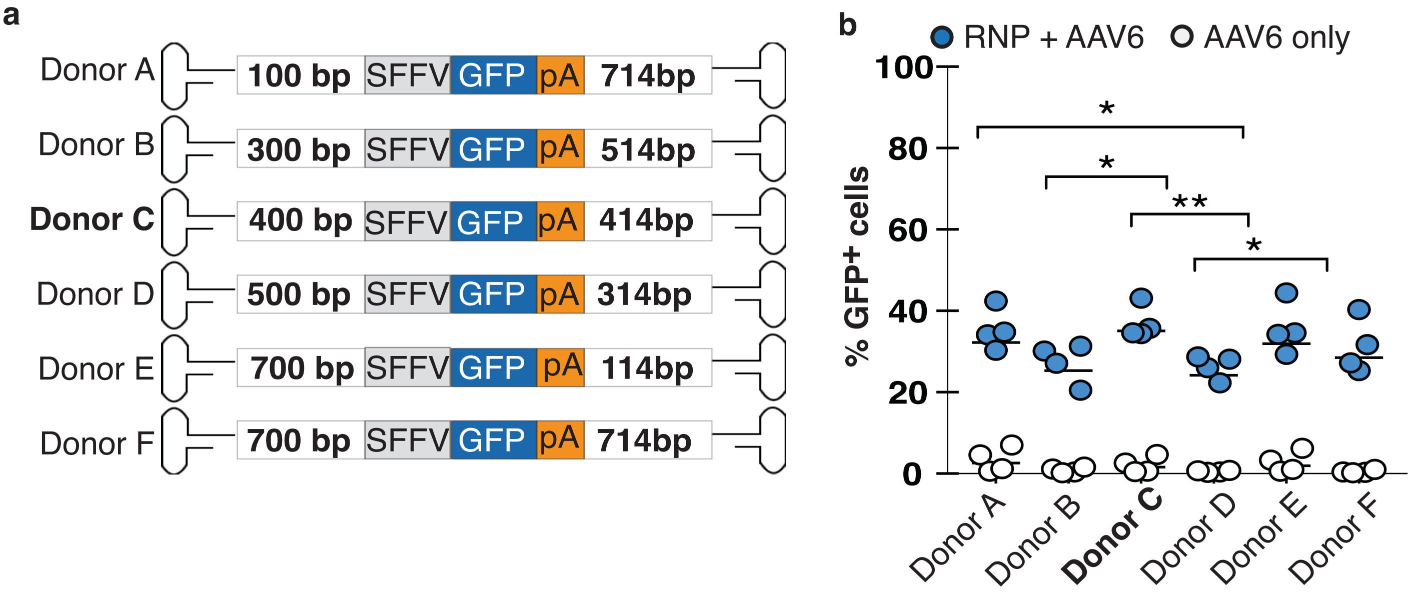

Supplementary Figure 4. IL2RG homology arms length characterization. (**a**) Schematics of various symmetric and asymmetric arms of homology flanking a SFFV GFP cassette. (**b**) Targeting integration frequencies (% HR) quantified by FACS analysis. Donor A vs Donor D *p-value = 0.0204; Donor B vs Donor C *p-value = 0.0226 ; Donor C vs Donor D **p-value = 0.0055; Donor D vs Donor E *p-value= 0.0361 (unpaired t-test). Median is shown. $CD34^+$ HSPCs used in this experiment were derived from two different fresh UCB or mPB donors. n = 4 healthy, male donors. UCB, umbilical cord blood, mPB, mobilized peripheral blood.

Supplementary Figures

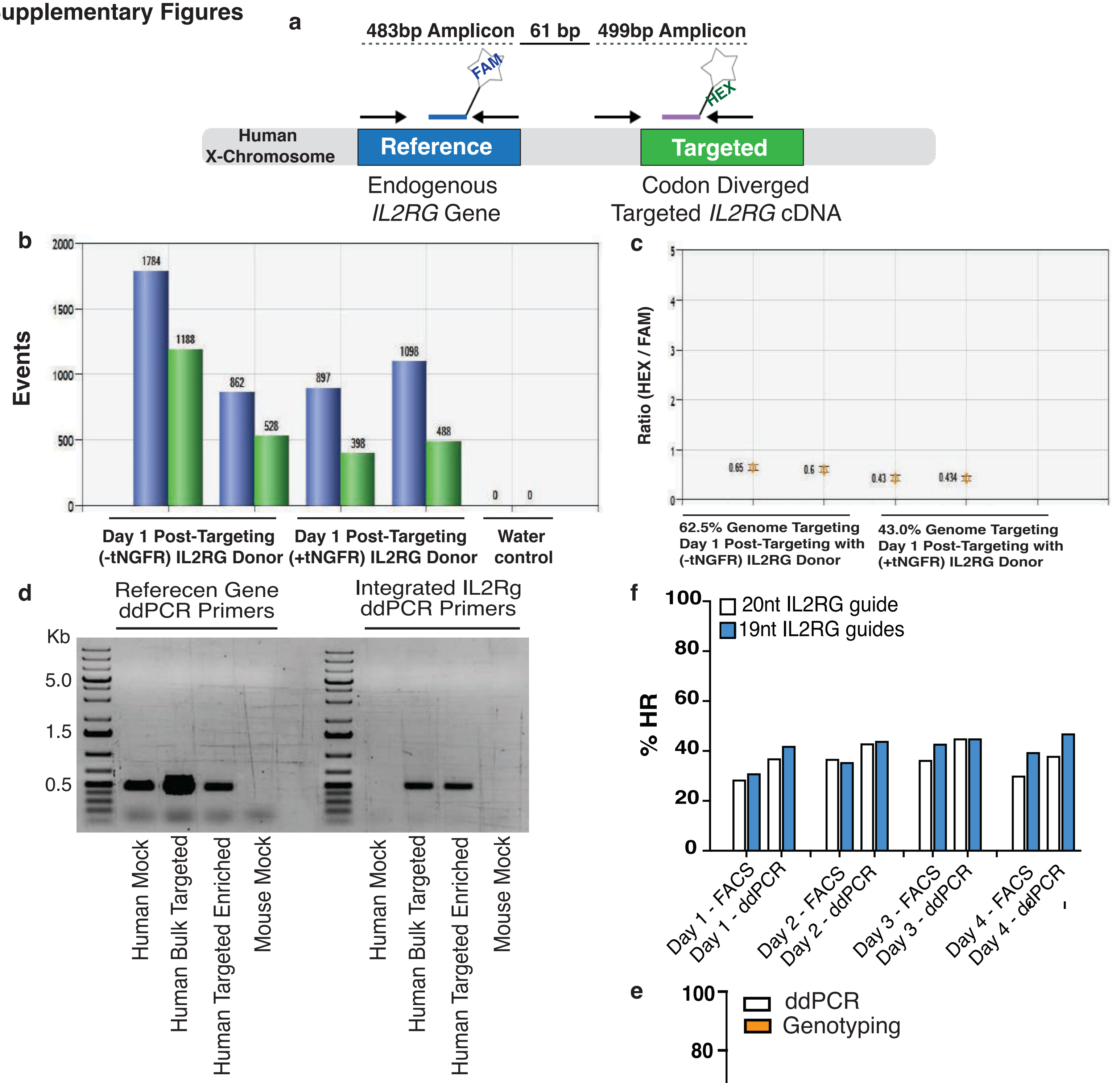

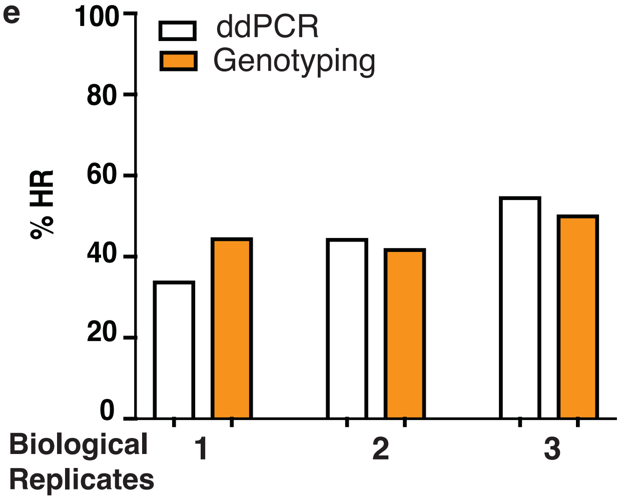

Supplementary Figure 5. IL2RG specific digital droplet PCR (ddPCR) assay.(**a**) Schematic representation of the IL2RG specific ddPCR primers-probe design. (**b**) Positive droplets generated for the reference (FAM labeled - blue) and integrate (HEX labeled - green) IL2RG PCR amplicons. Genome targeting results using -tNGFR or +tNGFR IL2RG cDNA targeted donors at 24h post rAAV6 transduction. (**c**) Ratio of integrated (HEX) to reference (FAM). Male derived genomic DNA contains only one allele of the human X-chromosome allowing for the ratio of the fluorescence signal to be a direct measurement of the levels of genome targeting. (**d**) Specificity of the ddPCR primer-probe set. (**e**) Comparison of ddPCR analysis of bulk IL2RG cDNA targeted male derived $CD34^{+}$ HSPCs and genotype of single cell sorted methylcellulose assay from the bulk population; n = 3 (biological replicates). (**f**) Comparison of ddPCR and FACS analysis of targeted SFFV-GFP cassette targeted into IL2RG locus of male derived $CD34^{+}$ HSPCs. Time course day 1 through 4-post targeting is shown for full length (20nt) and truncated (19nt) IL2RG guide.

Supplementary Figures

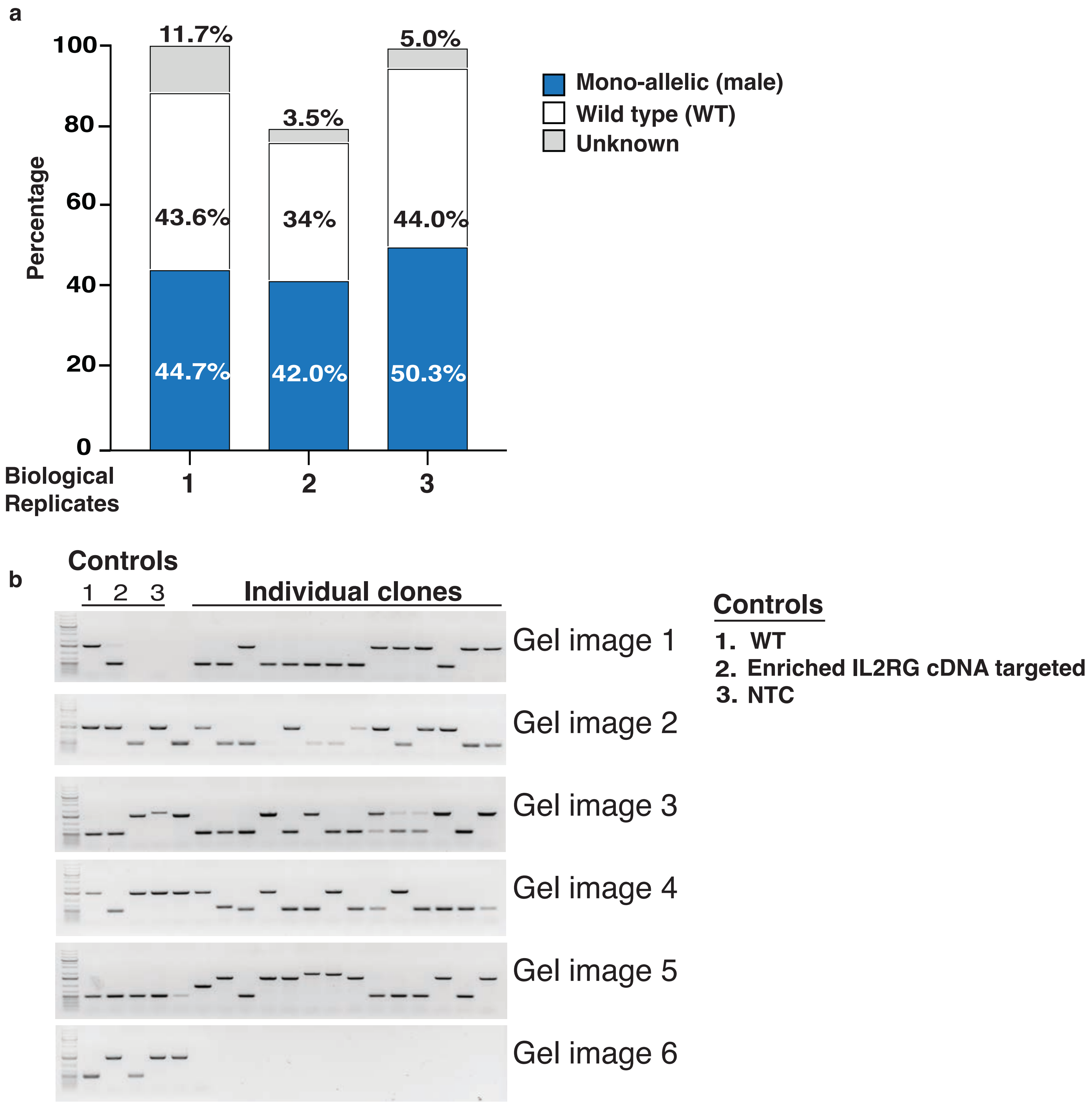

Supplementary Figure 6. Methylcellulose derived colonies and genotyping analysis of IL2RG cDNA targeted CD34+ HSPC single cells. (**a**) Quantification of percent allelic targeting. Genotyping results derived from day 2 post IL2RG genome targeting, n=3 biological replicates, male derived frozen mobilized PB CD34+ HSPCs. (**b**) Representative genotyping gel images. Colonies obtained at 14 days from individual wells of methylcellulose plates are scored and genotypes using a 3-primer PCR approach. Shown are genotyping results of biological replicate #2 from (**a**). WT - wild type; NTC - no template control; PB - peripheral blood.

Supplementary Figures

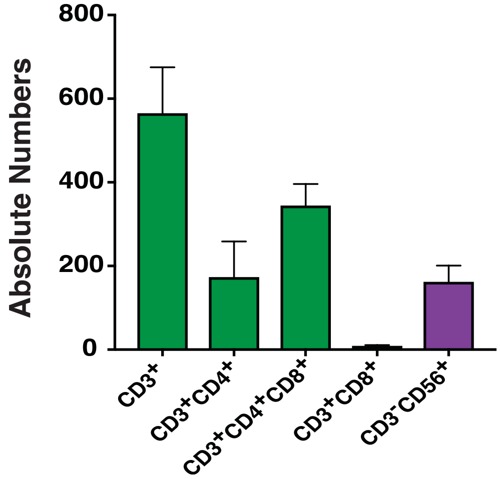

Supplementary Figure 7. In vitro IL2RG gene correction of SCID-X1 derived CD34$^+$ HSPCs. Shown are absolute numbers of pre-T cells, T-cells and NK cells derived at week one following induction of delta like 1 ligand (dll1) using doxycycline, from **Figure 1e**. n = 23 wells, mean ± s.e.m.

Supplementary Figures

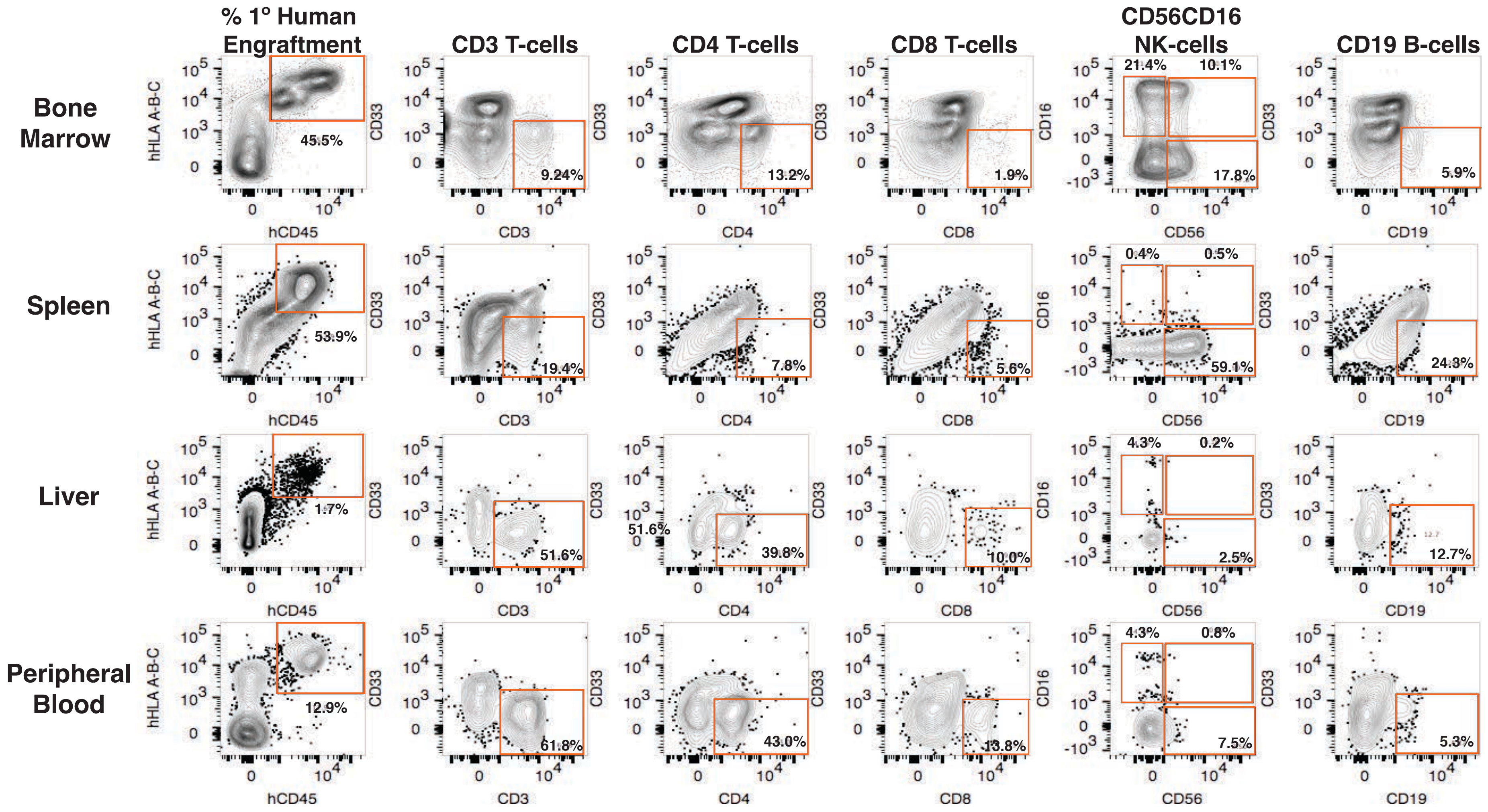

Supplementary Figure 8. Representative FACS plots for lymphoid lineage analysis at week 16 post intra-hepatic (IH) primary (1°) engraftment of IL2RG targeted $CD34^+$ HSPCs into new born NSG mice. Analysis is shown from a mouse with high (45.5%) human engraftment levels ($hCD45^+$ $hHLA\text{-}ABC^+$).

Supplementary Figures

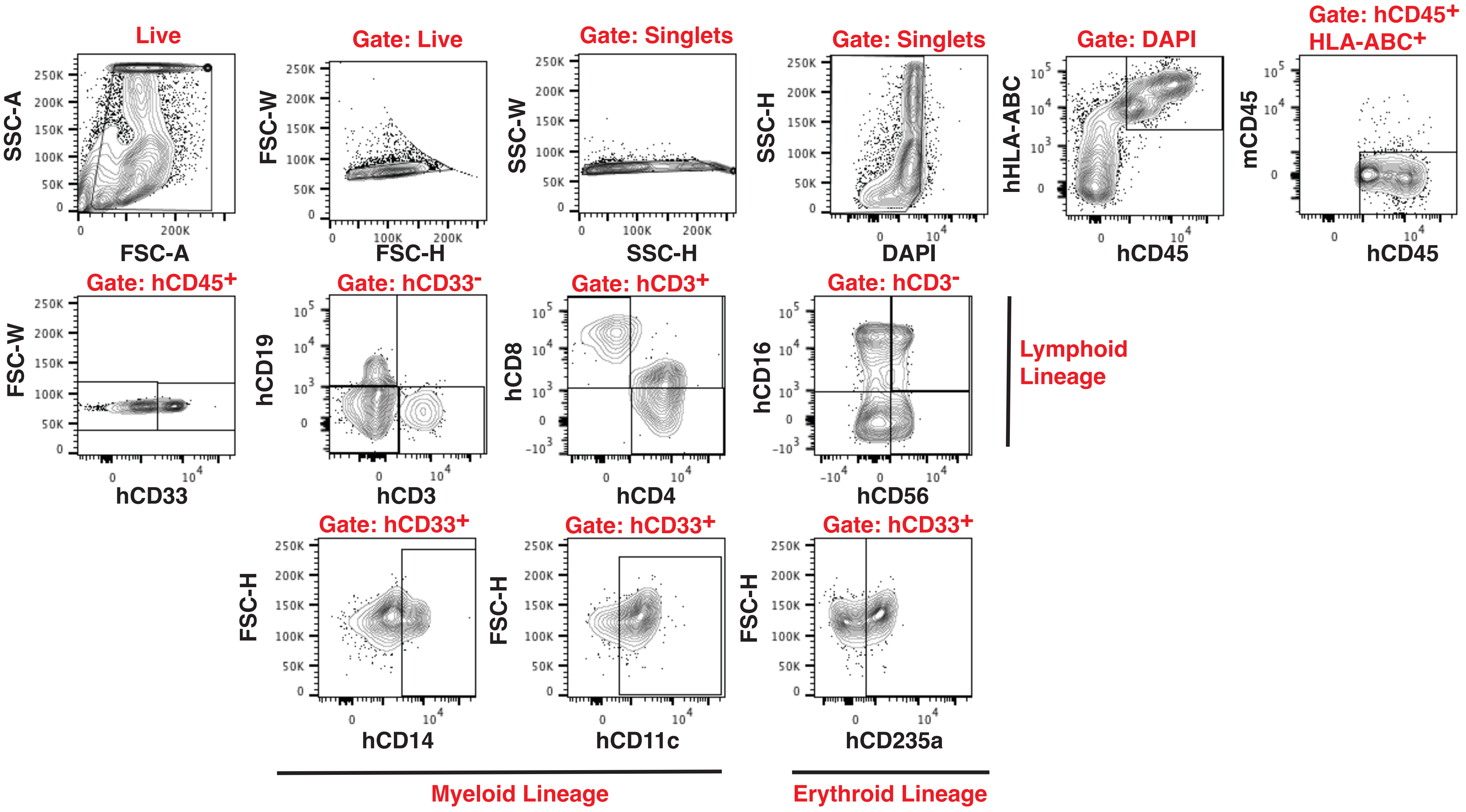

Supplementary Figure 9. Gating strategy for multilineage analysis. The following gating strategy was used to analyze multilineage development in vitro (Figure1e) and in vivo (Figure 2c-f, Figure 3a-c, Figure 4e-g, Supplementary Figure 11, Supplementary Figure 12, Supplementary Figure 13, Supplementary Figure 14).

Supplementary Figures

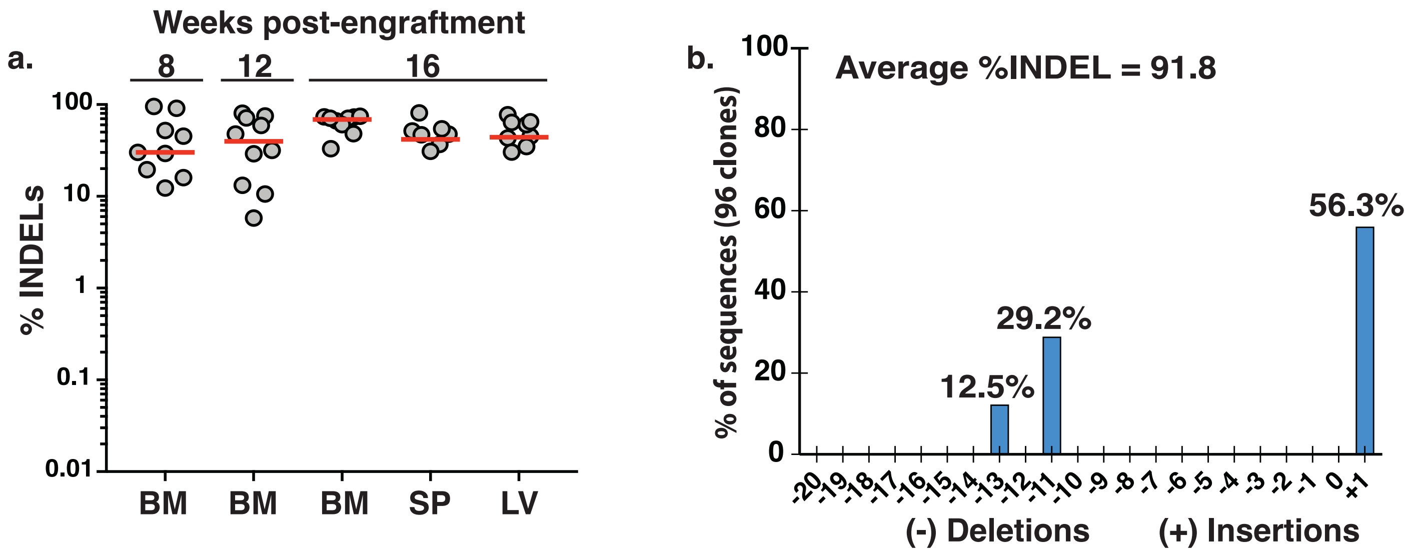

Supplementary Figure 10. On-target INDEL spectrum analysis of truncated (19nt) IL2RG sg-1 in $CD34^+$ HSPCs. (**a**) 1.0 x 10^5 $CD34^+$ HSPCs derived from male, frozen mobilized PB source nucleofected with RNP system at 5:1 molar ratio. Percent INDELs determined by TIDE analysis at 8, 12 and 16 weekspost 1° IH engraftment into NSG pups. (**b**) INDEL spectrum characterization generated by truncated 19nt IL2RG sg-1 at day 4-post nucleofection of male derived $CD34^+$ HSPCs. Analysis was carried out at clonal level: 96 clones obtained from TOPO cloning bulk RNP sample. IL2RG alleles obtained from each clone were sequenced and their INDELs' distribution determined by TIDE analysis.

Supplementary Figures

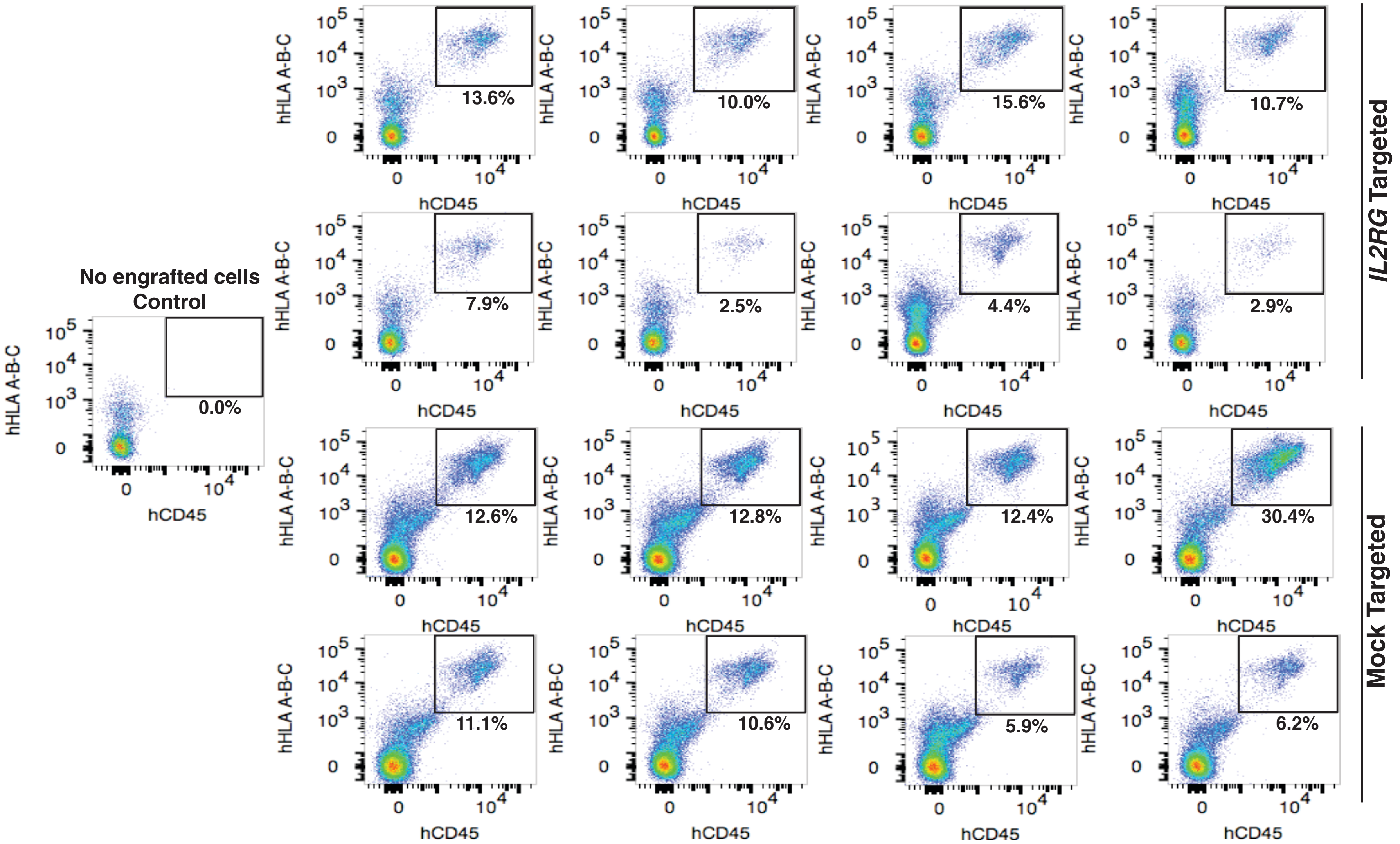

Supplementary Figure 11. FACS plots of secondary (2°) human engraftment levels (hCD45$^+$ hHLA-ABC$^+$). Secondary engraftment was carried out from total BM derived from primary (1°) IH IL2RG and mock targeted CD34$^+$ HSPCs. BM, bone marrow.

Supplementary Figures

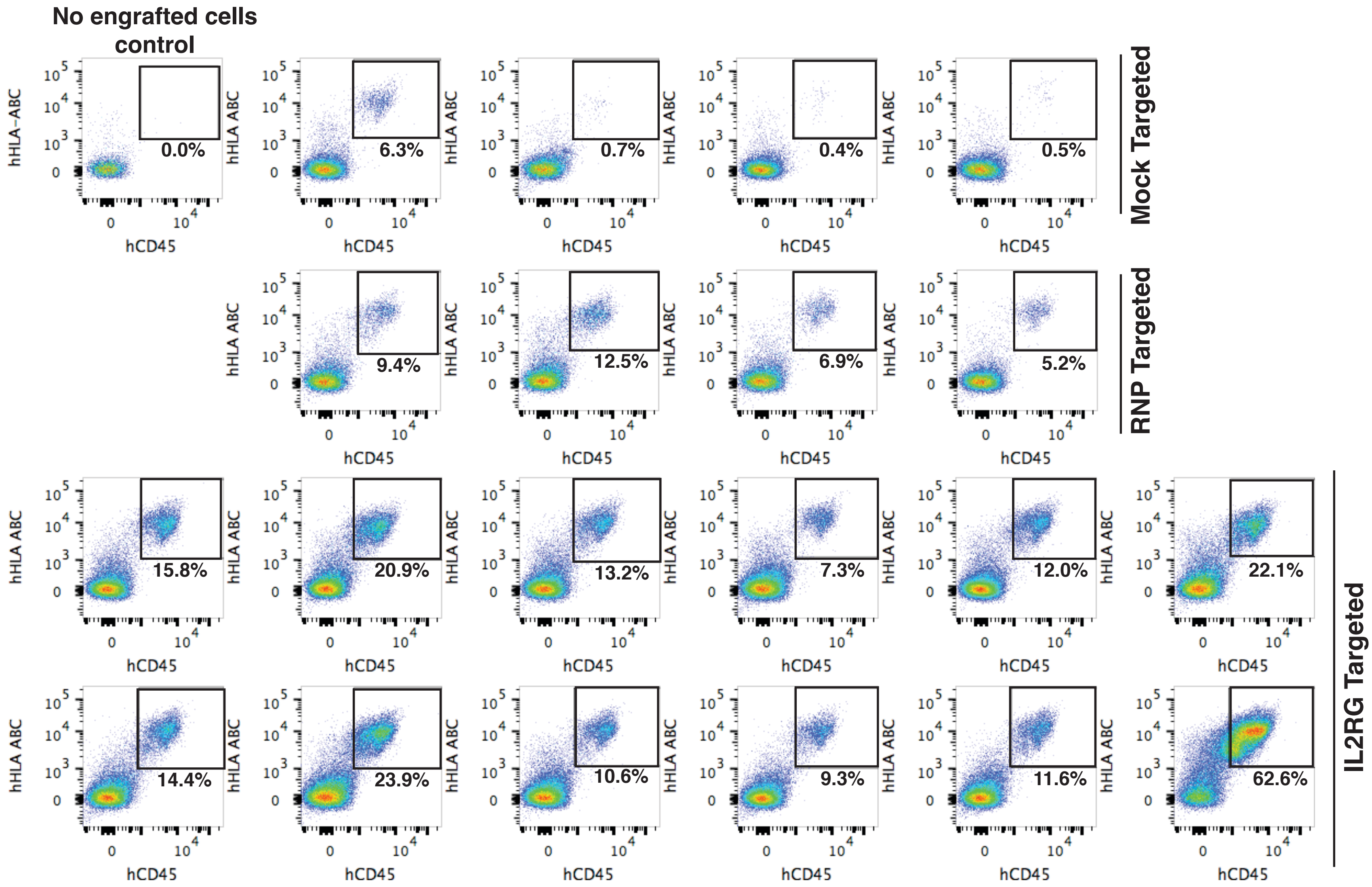

Supplementary Figure 12. FACS plots of 2° human engraftment levels ($hCD45^{+}$ $hHLA\text{-}ABC^{+}$) from total BM of mice injected IF with IL2RG or mock targeted $CD34^{+}$ HSPCs. 5 x 10^5 purified $CD34^{+}$HSPCs from total BM of mock or IL2RG targeted engrafted mice were injected IF into sub-lethally irradiated adult NSG mice. The observed low levels of engraftment in 3 out of 4 mice that received mock treated cells were due to fluid backflow during the IF injection procedure. IF: intra-femoral.

Supplementary Figures

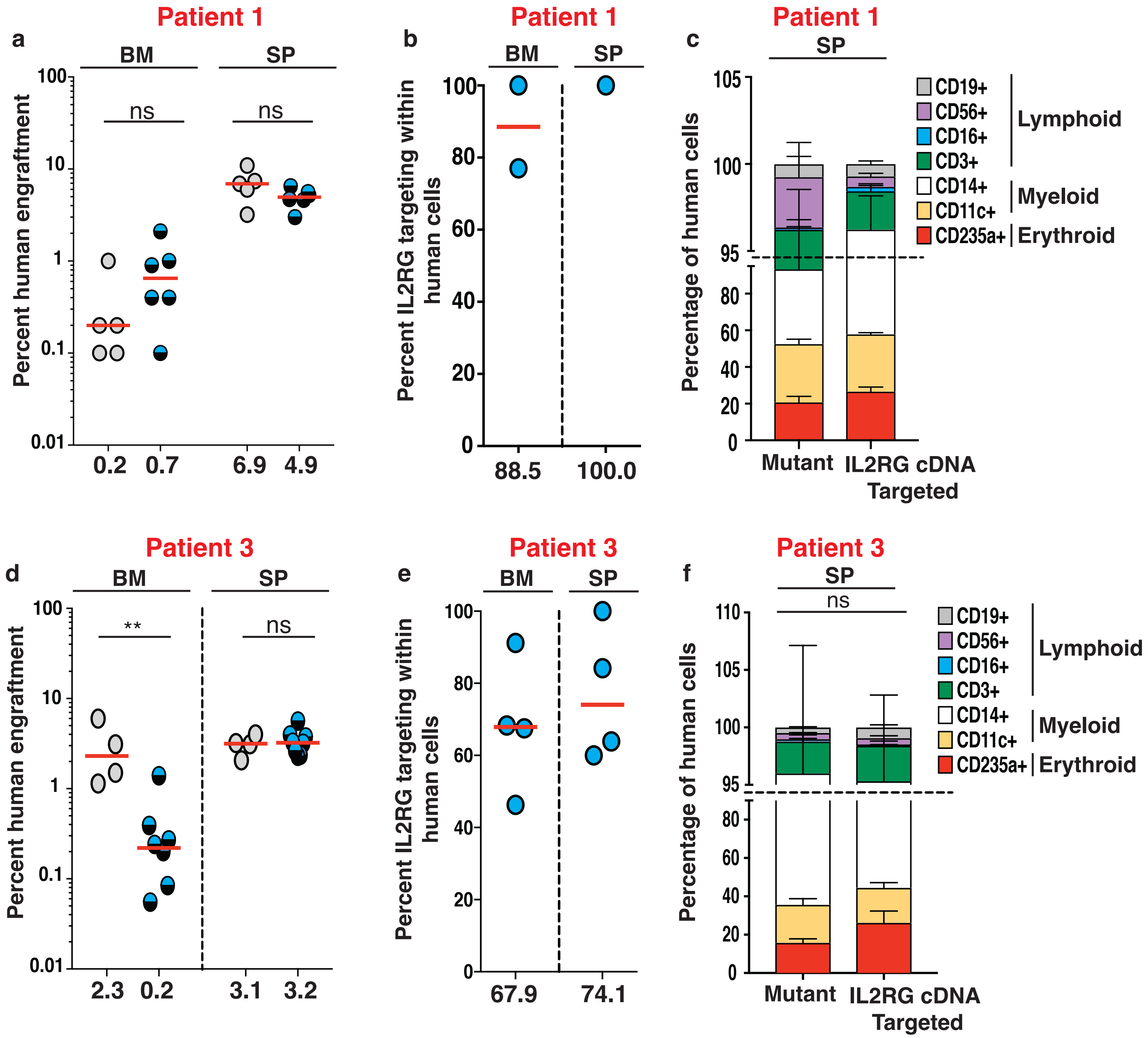

Supplementary Figure 13. Primary human engraftment of SCID-X1 patient derived CD34$^+$ HSPCs. (**a**) Human engraftment of mutant or IL2RG targeted HSPCs in bone marrow (BM, n = 5) or spleen (SP, n = 6) 20 weeks after transplant (median plotted). (**b**) ddPCR quantification of levels of IL2RG codon-optimized cDNA present in BM (n=2) and SP (n=1) samples. (**c**) Percent composition of lymphoid, myeloid and erythroid present in SP 20 weeks post-transplant. (**d**) Same as (**a**) using SCID-X1 patient 3 derived CD34$^+$ HSPCs mutant cells (n = 4) and IL2RG targeted cells (n=7) 17 weeks after transplant. Multiple t-test, Holm-Sidak test, median plotted. (**e**) Same as (**b**), n = 4. (**f**) Same as (**c**) **p-value = 0.0073, ns, not significant.

Supplementary Figures

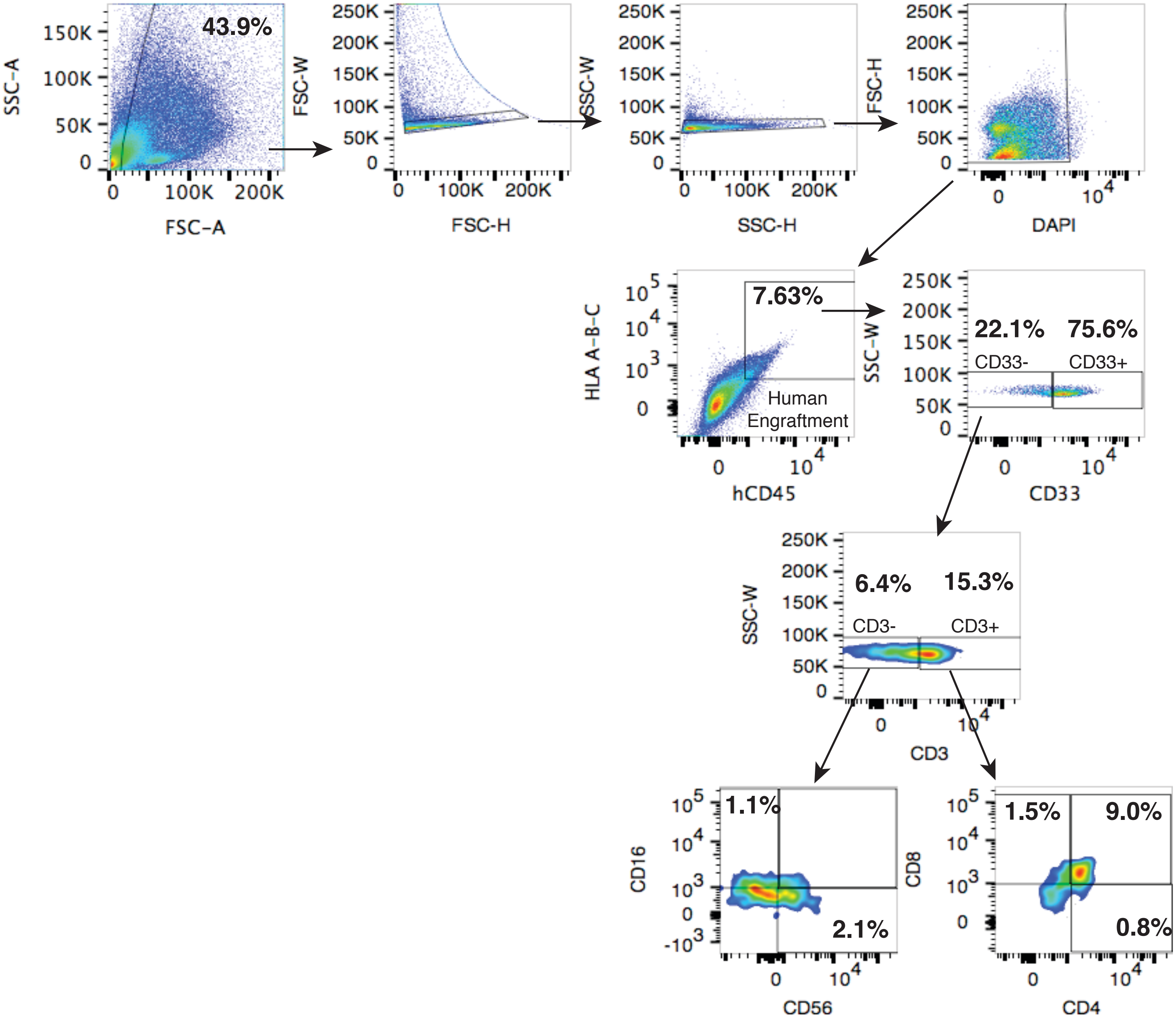

Supplementary Figure 14. Lymphoid lineage analysis of IL2RG cDNA targeted SCID-X1 patient 2 derived CD34$^+$HSPCs. Representative FACS analysis of spleen sample derived from one NSG mouse at week 16 post engraftment with IL2RG cDNA targeted mobilized PB CD34$^+$HSPCs. PB, peripheral blood.

Supplementary Figures

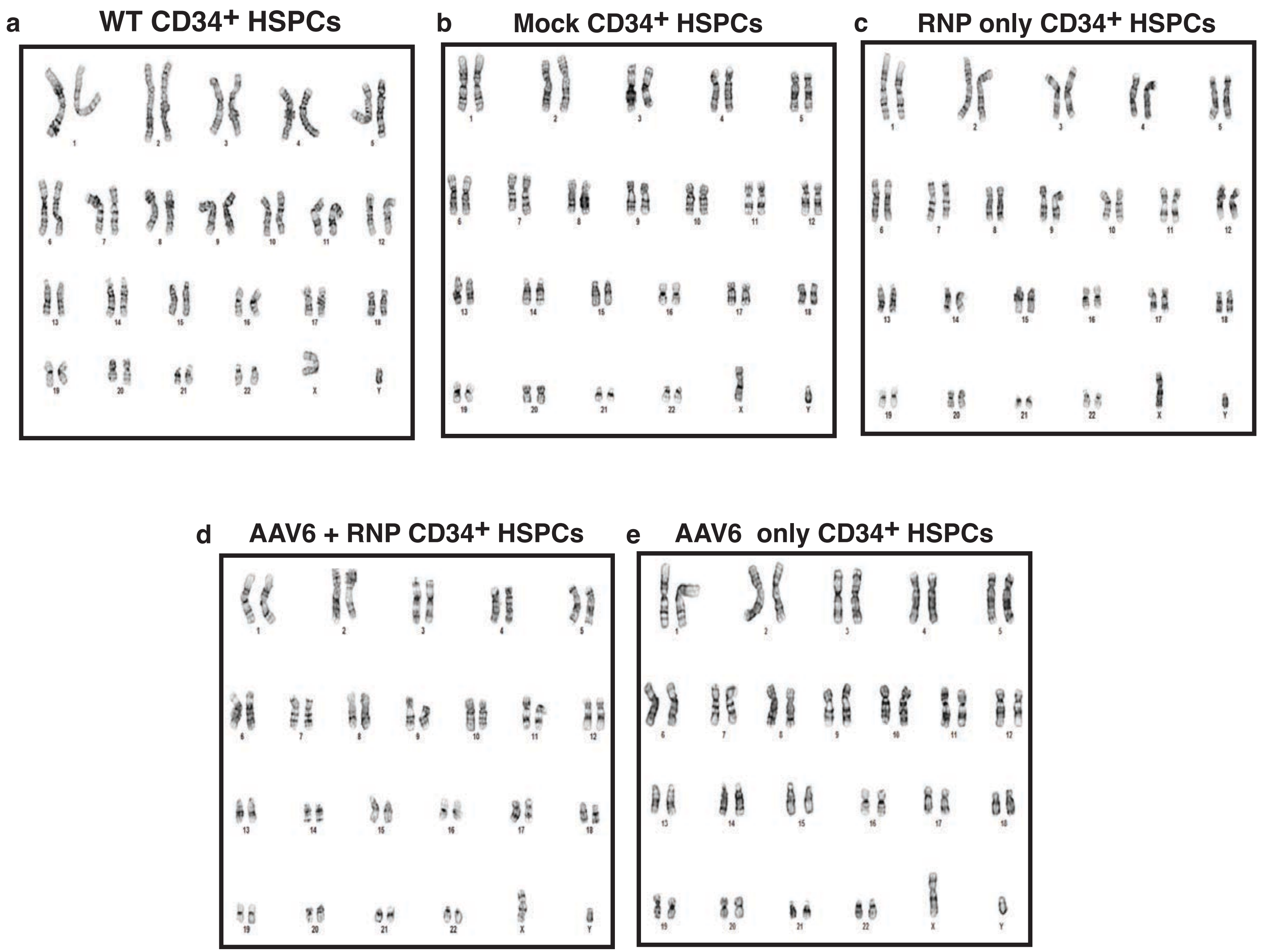

Supplementary Figure 15. Karyotype analysis of IL2RG cDNA genome edited and genome targeted cord blood derived $CD34^{+}$ HSPCs. 5.0×10^{5} cells were nucleofected at day 2 post ex-vivo cell culturing with RNP at 5:1 molar ratio. Conditions (**d**) and (**e**) received rAAV6 with -tNGFR IL2RG clinical donor at an MOI of 200,000 vgc/ul. Day 2 post transduction, cells were collected and prepared the same day for karyotype analysis. 20 cells were analyzed per condition. Conditions (**c**) and (**d**) and conditions (**d**) and (**e**) show that a combined 40/40 cells treated with RNP or with rAAV6, respectively did not produced cells with chromosomal abnormalities. vgc: viral genome copies.

Supplementary Figures

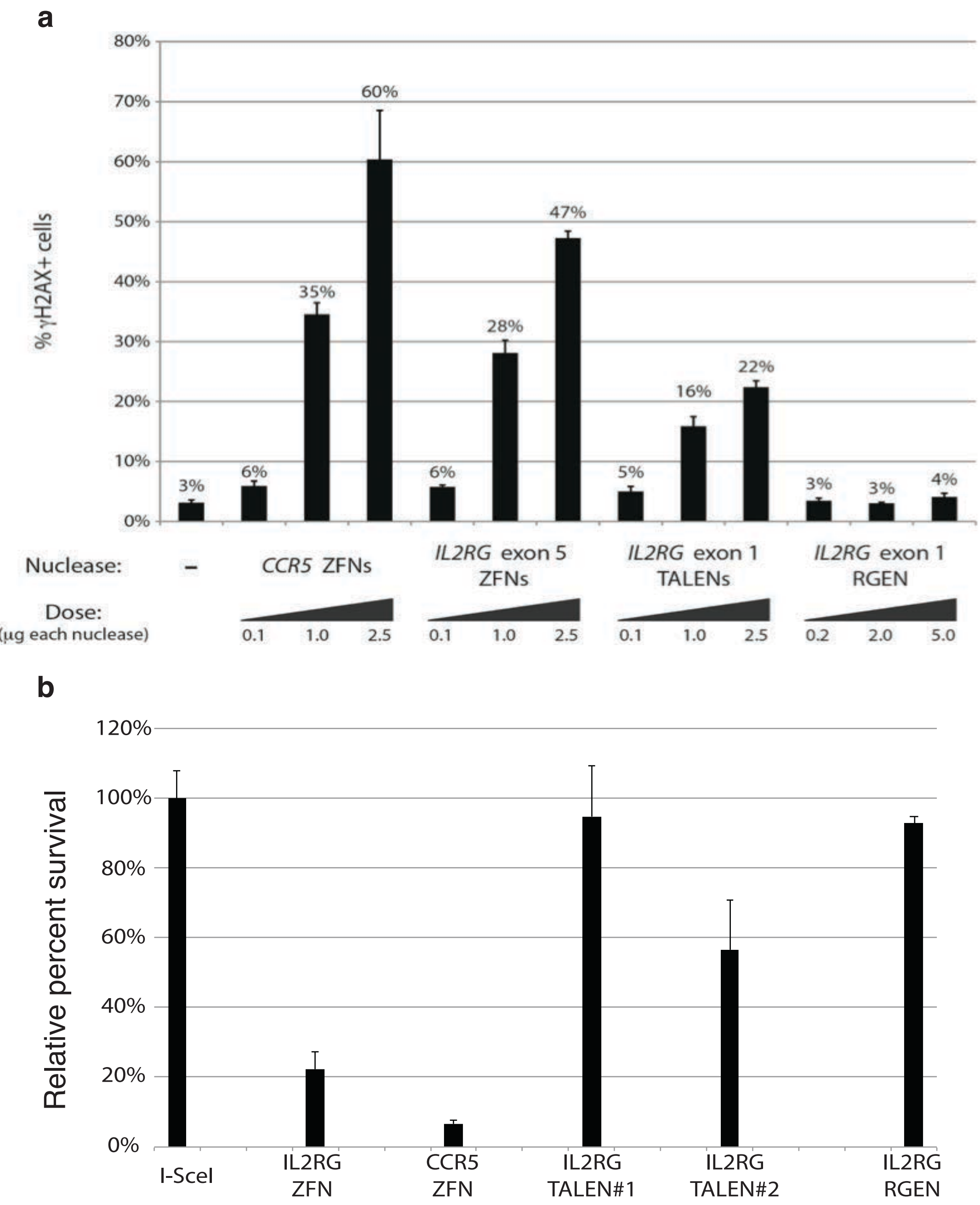

Supplementary Figure 16. Genotoxicity of IL2RG exon 1 TALENs, IL2RG exon 5 ZFNs, CCR5 ZFNs, and an IL2RG exon 1 RGEN (CRISPR-Cas9). (**a**) γH2AX assay. Genotoxicity assay measuring DNA damage induced by different classes of engineered nucleases by assessing the phosphorylation of histone H2AX, a marker of DSB formation, in K562 cells. Percentage γ$H2AX^+$ cells was measured by flow cytometry 48h post-nucleofection. (**b**) Relative cell survival assay. Levels of genotoxicity induced by different classes of engineered nucleases in 293T cell line. Cells were nucleofected with GFP plasmid DNA and genome wide off-target activity of each nuclease was determined by FACS analysis as percent GFP^+ cells relative to I-SceI control. Bars: n = 3, mean ± s.d.

Table S1 – sgRNA Guides sequence for IL2RG exon 1

sgRNA Guide ID	sgRNA Guide Sequence	PAM sequence
**sgRNA 1**	5' TGGTAATGATGGCTTCAACA 3'	TGG
**sgRNA 2**	5' GGGCAGCTGCAGGAATAAGA 3'	GGG
**sgRNA 3**	5' AGGGATGTGAATGGTAATGA 3'	TGG
**sgRNA 4**	5' TTCAGCCCCACTCCCAGCAG 3'	GGG
**sgRNA 5**	5'ATTCCTGCAGCTGCCCCTGC 3'	TGG
**sgRNA 6**	5' CGACAATTCTGACGCCCAAT 3'	GGG
**sgRNA 7**	5' AGCTGCCCCTGCTGGGAGTG 3'	GGG

Table S2 – Primers and probes for ddPCR based assay

Primer Name	Primer Sequence	Amplicon size (bp)
ddPCR-cDNA-F	5' GGGTGACCAAGTCAAGGAAG 3'	499
ddPCR-cDNA-R	5' GATGGTGGTATTCAAGCCGA 3'	
ddPCR-cDNA-Probe	5' CAAGCGCCATGTTGAAACCCAGCCTGCCC 3'	
ddPCR-Reference-F	5' GGGAAGGTAAAACTGGCAAC 3'	483
ddPCR-Reference-R	5' GGGCACATATACAGCTGTCT 3'	
ddPCR-Reference-Probe	5' CCTCGCCAGTCTCAACAGGGACCCAGC 3'	
ddPCR-GFP-F	5' AAGGGGGAGGATTGGGAAG 3'	502
ddPCR-GFP-R	5' TCAGAAGGAGGAGGCCAAG 3'	
ddPCR-GFP-Probe	5' GCATGCTGGGGATGCGGTGGGC 3'	
