# Supplementary material for: Gene correction for SCID-X1 in long-term hematopoietic stem cells
Source: Nat Commun. 2019 Apr 9;10:1634. doi: 10.1038/s41467-019-09614-y (PMC6456568; doi:10.1038/s41467-019-09614-y)
Supplement: Supplementary file 2 — Reporting Summary [file 41467_2019_9614_MOESM2_ESM.pdf]

natureresearch

Corresponding author(s): *Double-blind peer review submissions: write DBPR and your manuscript number here instead of author names.*

Reporting Summary

Nature Research wishes to improve the reproducibility of the work that we publish. This form provides structure for consistency and transparency in reporting. For further information on Nature Research policies, see Authors & Referees and the Editorial Policy Checklist.

Statistical parameters

When statistical analyses are reported, confirm that the following items are present in the relevant location (e.g. figure legend, table legend, main text, or Methods section).

n/a	Confirmed	
☐	☒	The exact sample size (*n*) for each experimental group/condition, given as a discrete number and unit of measurement
☐	☒	An indication of whether measurements were taken from distinct samples or whether the same sample was measured repeatedly
☐	☒	The statistical test(s) used AND whether they are one- or two-sided<br>*Only common tests should be described solely by name; describe more complex techniques in the Methods section.*
☒	☐	A description of all covariates tested
☐	☒	A description of any assumptions or corrections, such as tests of normality and adjustment for multiple comparisons
☐	☒	A full description of the statistics including central tendency (e.g. means) or other basic estimates (e.g. regression coefficient) AND variation (e.g. standard deviation) or associated estimates of uncertainty (e.g. confidence intervals)
☐	☒	For null hypothesis testing, the test statistic (e.g. *F*, *t*, *r*) with confidence intervals, effect sizes, degrees of freedom and *P* value noted<br>*Give P values as exact values whenever suitable.*
☒	☐	For Bayesian analysis, information on the choice of priors and Markov chain Monte Carlo settings
☒	☐	For hierarchical and complex designs, identification of the appropriate level for tests and full reporting of outcomes
☒	☐	Estimates of effect sizes (e.g. Cohen's *d*, Pearson's *r*), indicating how they were calculated
☐	☒	Clearly defined error bars<br>*State explicitly what error bars represent (e.g. SD, SE, CI)*

Our web collection on statistics for biologists may be useful.

Software and code

Policy information about availability of computer code

Data collection	BD FACS Diva software v 8.0, QuantaSoft software, ABI Prism 7900HT Sequence detection system, BD Accuri C6 Plus software, Nikon Eclipse TS100, Illumina MiSeq
Data analysis	Prism 7 GraphPad Software, FlowJo, QuantaSoft, COSMID, TIDE (Tracking of Indels by Decomposition)

For manuscripts utilizing custom algorithms or software that are central to the research but not yet described in published literature, software must be made available to editors/reviewers upon request. We strongly encourage code deposition in a community repository (e.g. GitHub). See the Nature Research guidelines for submitting code & software for further information.

Data

Policy information about availability of data

All manuscripts must include a data availability statement. This statement should provide the following information, where applicable:

- Accession codes, unique identifiers, or web links for publicly available datasets
- A list of figures that have associated raw data
- A description of any restrictions on data availability

The authors declare that data supporting the findings of this study are available within the paper and its supplementary information files. The DNA sequence of the codon optimized IL2RG cDNA used in this study is available from the corresponding author upon reasonable request.

Field-specific reporting

Please select the best fit for your research. If you are not sure, read the appropriate sections before making your selection.

☒ Life sciences ☐ Behavioural & social sciences ☐ Ecological, evolutionary & environmental sciences

For a reference copy of the document with all sections, see nature.com/authors/policies/ReportingSummary-flat.pdf

Life sciences study design

All studies must disclose on these points even when the disclosure is negative.

Sample size	The minimum sample size was determined by the amount of mice needed to parse out statistical significance. Statistical analyses used in this study are: Welch's t-test, Kruskal-Wallis test, unpaired t-test, Multiple t-test, Holm-Sidak test.
Data exclusions	Any exclude data was either due to death of mouse or loss of animal organ prior to a complete analysis was carried out.
Replication	All experiments were replicated with an n=3. All attempts at replicating the experiments were successful with the exception of in vivo reconstitution of immune system from two additional different SCID-X1 patient derived CD34+ HSPCs. This was due to due to the overall low levels of human engraftment in transplanted mice.
Randomization	Cord blood derived CD34+ HSPCs were derived exclusively from male healthy donors since SCID-X1 disease affects only male infants. The type of SCID-X1 pathogenic mutation used in the study was random and based on the availability of such samples.
Blinding	Blinding was not relevant to this study.

Reporting for specific materials, systems and methods

Materials & experimental systems

n/a	Involved in the study
☐	☒ Unique biological materials
☐	☒ Antibodies
☐	☒ Eukaryotic cell lines
☒	☐ Palaeontology
☐	☒ Animals and other organisms
☒	☐ Human research participants

Methods

n/a	Involved in the study
☒	☐ ChIP-seq
☐	☒ Flow cytometry
☒	☐ MRI-based neuroimaging

Unique biological materials

Policy information about availability of materials

Obtaining unique materials	Cord blood derived CD34+ HSPCs were obtained from healthy male donors, from Stanford University, under informed consent. De-identified SCID-X1 patient derived frozen mobilized peripheral blood CD34+ HSPCs (IRB-approved protocol 94-I-0073) were obtained from NIH (National Institutes of Allergy and Infectious Disease) following a signed MTA.

Antibodies

Antibodies used	Live/Dead blue dead cell staining kit for UV (ThermoFisher Scientific, cat no. L23105), CD3 PerCP/Cy5.5 (HiT3A, BioLegend), CD4 BV650 (OKT4, BioLegend), CD8 APC (HiT8a, BioLegend), CD11c BV605 (3.9, BioLegend), CD14 BV510 (M5E2, BioLegend), CD19 FITC (HIB19, BioLegend), CD33 AF-300 (WM53, BDPharmingen), CD45 BV786 (BDPharminge), CD56 PE (MEM-188 BioLegend), CD235a PE-Cy7 (HI264, BioLegend), CD271 (tNGFR) CF-594 (C40-1457, BD Horizon), CD3 PE (UCHT1, BioLegend), CD271 (tNGFR) APC (ME20.4, Biolegend), pSTAT5 AF-488 (pY694, BD Bioscience), isotype control (BD Biosciences).
Validation	For each antibody used in this study a PDF link containing a detailed description of its generation, use, validation and related cited work is provided.<br>pSTAT5 AF-488 (pY694, BD Bioscience): http://www.bdbiosciences.com/us/applications/research/intracellular-flow/intracellular-antibodies-and-isotype-controls/anti-human-antibodies/alexa-fluor-488-anti-stat5-py694-47stat5py694/p/562075<br>CD271 (tNGFR) APC (ME20.4, Biolegend): https://www.biolegend.com/de-at/global-elements/pdf-popup/apc-anti-human-cd271-ngfr-antibody-6877?filename=APC%20anti-human%20CD271%20NGFR%20Antibody.pdf&pdfgen=true<br>CD3 PE (UCHT1, BioLegend): https://www.biolegend.com/de-at/global-elements/pdf-popup/pe-anti-human-cd3-antibody-865?

filename=PE%20anti-human%20CD3%20Antibody.pdf&pdfgen=true
CD271 (tNGFR) CF-594 (C40-1457, BD Horizon): https://www.bdbiosciences.com/us/applications/research/stem-cell-research/mesenchymal-stem-cell-markers-bone-marrow/human/positive-markers/pe-cf594-mouse-anti-human-cd271-c40-1457/p/563452
CD235a PE-Cy7 (HI264, BioLegend): https://www.biolegend.com/de-at/global-elements/pdf-popup/pe-cy7-anti-human-cd235a-glycophorin-a-antibody-9003?filename=PECy7%20anti-human%20CD235a%20Glycophorin%20A%20Antibody.pdf&pdfgen=true
CD56 PE (MEM-188 BioLegend): https://www.biolegend.com/de-at/global-elements/pdf-popup/pe-anti-human-cd56-ncam-antibody-1605?filename=PE%20anti-human%20CD56%20NCAM%20Antibody.pdf&pdfgen=true
CD45 BV786 (BDPharminge): https://www.bdbiosciences.com/us/reagents/research/antibodies-buffers/immunology-reagents/anti-mouse-antibodies/cell-surface-antigens/bv786-rat-anti-mouse-cd45-30-f11/p/564225
CD33 AF-300 (WM53, BDPharmingen): http://www.bdbiosciences.com/us/applications/research/stem-cell-research/hematopoietic-stem-cell-markers/human/negative-markers/purified-mouse-anti-human-cd33-wm53-also-known-as-wm-53/p/555449
CD19 FITC (HIB19, BioLegend): https://www.biolegend.com/de-at/global-elements/pdf-popup/fitc-anti-human-cd19-antibody-717?filename=FITC%20anti-human%20CD19%20Antibody.pdf&pdfgen=true
CD14 BV510 (M5E2, BioLegend): https://www.biolegend.com/de-at/global-elements/pdf-popup/brilliant-violet-510-anti-human-cd14-antibody-8001?filename=Brilliant%20Violet%20510%20anti-human%20CD14%20Antibody.pdf&pdfgen=true
CD11c BV605 (3.9, BioLegend): https://www.biolegend.com/de-at/global-elements/pdf-popup/brilliant-violet-605-anti-human-cd11c-antibody-8603?filename=Brilliant%20Violet%20605%20anti-human%20CD11c%20Antibody.pdf&pdfgen=true
CD8 APC (HiT8a, BioLegend): https://www.biolegend.com/de-at/global-elements/pdf-popup/brilliant-violet-650-anti-human-cd4-antibody-7786?filename=Brilliant%20Violet%20650%20anti-human%20CD4%20Antibody.pdf&pdfgen=true
Live/Dead blue dead cell staining kit for UV (ThermoFisher Scientific, cat no. L23105): https://www.thermofisher.com/order/catalog/product/L23105
PerCP/Cy5.5 anti-human CD3 Antibody : https://www.biolegend.com/en-us/global-elements/pdf-popup/percp-cy5-5-anti-human-cd3-antibody-5613?filename=PerCPCy55%20anti-human%20CD3%20Antibody.pdf&pdfgen=true
CD4 BV650 (OKT4, BioLegend): https://www.biolegend.com/de-at/global-elements/pdf-popup/brilliant-violet-650-anti-human-cd4-antibody-7786?filename=Brilliant%20Violet%20650%20anti-human%20CD4%20Antibody.pdf&pdfgen=true

Eukaryotic cell lines

Policy information about cell lines

Cell line source(s)
State the source of each cell line used.

Authentication
Describe the authentication procedures for each cell line used OR declare that none of the cell lines used were authenticated.

Mycoplasma contamination
Confirm that all cell lines tested negative for mycoplasma contamination OR describe the results of the testing for mycoplasma contamination OR declare that the cell lines were not tested for mycoplasma contamination.

Commonly misidentified lines
(See ICLAC register)
Name any commonly misidentified cell lines used in the study and provide a rationale for their use.

Animals and other organisms

Policy information about studies involving animals; ARRIVE guidelines recommended for reporting animal research

Laboratory animals
Human engraftment experimental design and mouse handling followed an approved Stanford University Administrative Panel on Lab Animal Care (APLAC). Immunodeficient NSG mice between the age of 3-4 days old or 6-8 weeks old, both male and females were used in the study.

Wild animals
The study did not involve wild animals.

Field-collected samples
The study did not involve field-collected samples.

Flow Cytometry

Plots

Confirm that:

☒ The axis labels state the marker and fluorochrome used (e.g. CD4-FITC).

☒ The axis scales are clearly visible. Include numbers along axes only for bottom left plot of group (a 'group' is an analysis of identical markers).

☒ All plots are contour plots with outliers or pseudocolor plots.

☒ A numerical value for number of cells or percentage (with statistics) is provided.

Methodology

Sample preparation
Level of human engraftment was assessed at weeks 8 and 12 using bone marrow aspirates and peripheral blood samples. At week 16 or later end point analysis was done from total bone marrow, spleen, liver and peripheral blood. For total bone marrow analysis, mouse bones were harvest from tibiae, femurs, sternum and spinal cord from each mouse and grinded using a mortar and pestle. Mononuclear cells (MNC) were purified using Ficoll gradient centrifugation (Ficoll-Paque Plus, GE Healthcare,

Sunnyvale, CA, USA) for 25 min at 2,000 x g, at room temperature. Spleen and liver samples were grinded against a 40uM mesh, transferred to a FACS tube and spun down at 300 x g for 5 min, at 4°C. Red blood cells were lysed following a 10-12 min incubation on ice with 500ul of 1x ACK lysis buffer (ThermoScientific, cat no. A1049201). Reaction was quenched and cells were washed with MACS buffer (2% - 5% FBS, 2mM EDTA and 1x PBS). Peripheral blood samples were treated with 500 ul of 2% Dextren and incubated at 37C for 30 min to 1h. 800 ul to 1ml of the top layer was transferred to a FACS tube, spun down at 300 x g, 5 min and red blood cells lysed as already described. Cells purified from all 4 sources were re-suspended in 50 ul MACS buffer, blocked, stained with LIVE/Dead staining solution and stained for 30 min at 4°C, dark with pre-determined antibody panel, described in material and methods section.

Secondary engraftments experiments were derived from both IH and from IF engrafted human cells. From the IH mock and IL2RG cDNA targeted engrafted mice, total bone marrow was collected at week 16 post-primary engraftment, MNC were purified using Ficoll gradient centrifugation and CD34+ cells were enriched using CD34+ microbeads (Miltenyi). Enriched cells were pooled from 5 mock treated cells and from 7 IL2RG cDNA targeted cells and cultured overnight in complete CD34+ media containing UM171 and SR1. Following overnight incubation, cellular count and viability was determined for mock treated cells to be 2.47x 106 cells at 85.5% viability and for IL2RG cDNA targeted cells was 4.8x106 cells at 84% viability. 3.5x105 mock treated cells and 5.0x105 IL2RG cDNA targeted cells were engrafted IF into 8 6-8 weeks old, irradiated NSG mice (4 males and 4 females).

Instrument	BD FACS Aria II Cell Sorter instrument, part number 650110.
Software	BD FACS Aria II DIVA software v8
Cell population abundance	Detailed post-sort analysis is provided in the supplemental material.
Gating strategy	A detailed gating strategy is provided in the supplemental material.

☒ Tick this box to confirm that a figure exemplifying the gating strategy is provided in the Supplementary Information.
